# Supplementary material for: Role of PLEXIND1/TGFβ Signaling Axis in Pancreatic Ductal Adenocarcinoma Progression Correlates with the Mutational Status of KRAS
Source: Cancers (Basel). 2021 Aug 11;13(16):4048. doi: 10.3390/cancers13164048 (PMC8393884; doi:10.3390/cancers13164048)
Supplement: Supplementary file 1 [file cancers-13-04048-s001.zip › cancers-1302602 Figure S8 Original Western Blots.pdf]

Figure 1 : Panel B

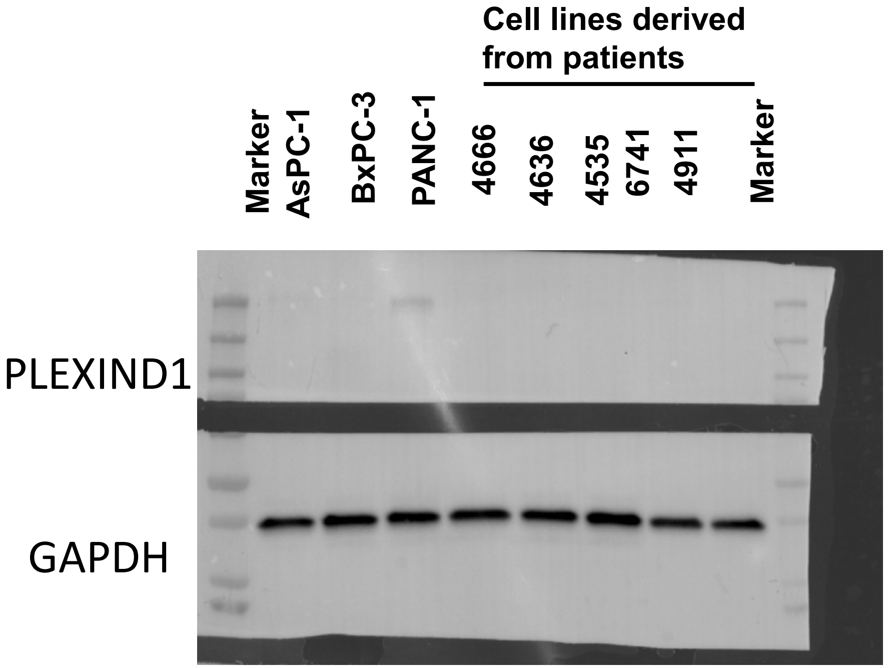

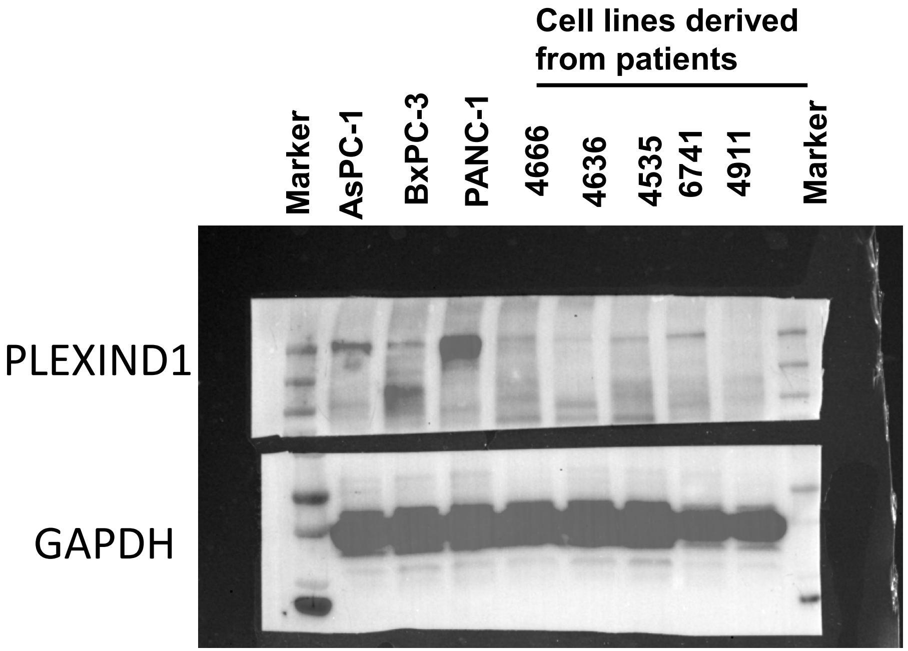

Figure 1 : Panel C

PANC-1

Marker 1 2

PLEXIND1

GAPDH

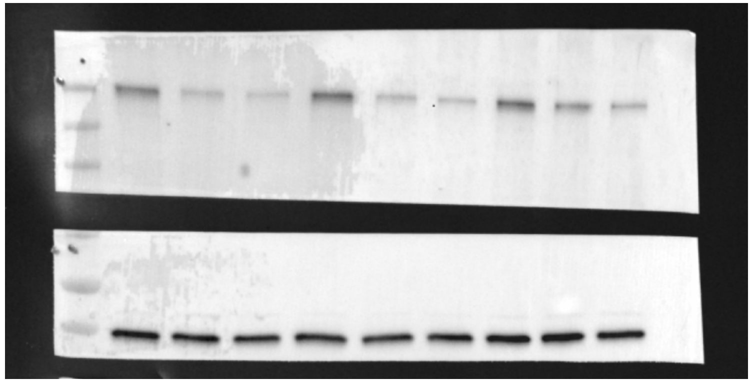

1: PANC-1 cont sh
2: PANC-1 PLEXIND1sh1

Figure 1 : Panel E

6 7

PLEXIND1

GAPDH

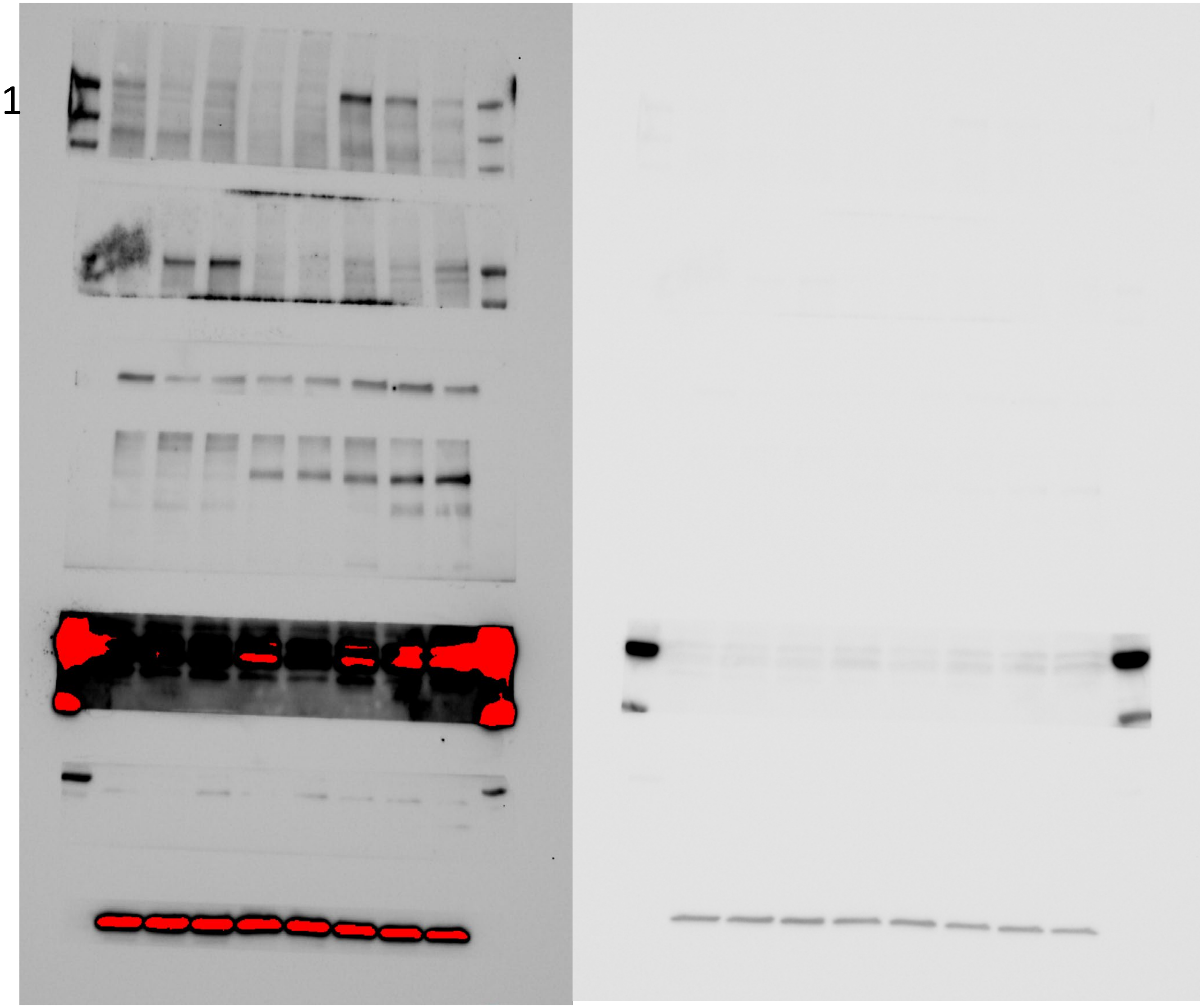

6: AsPC-1 cont sh
7: AsPC-1 PLEXIND1sh1

Figure: 2 Panel A

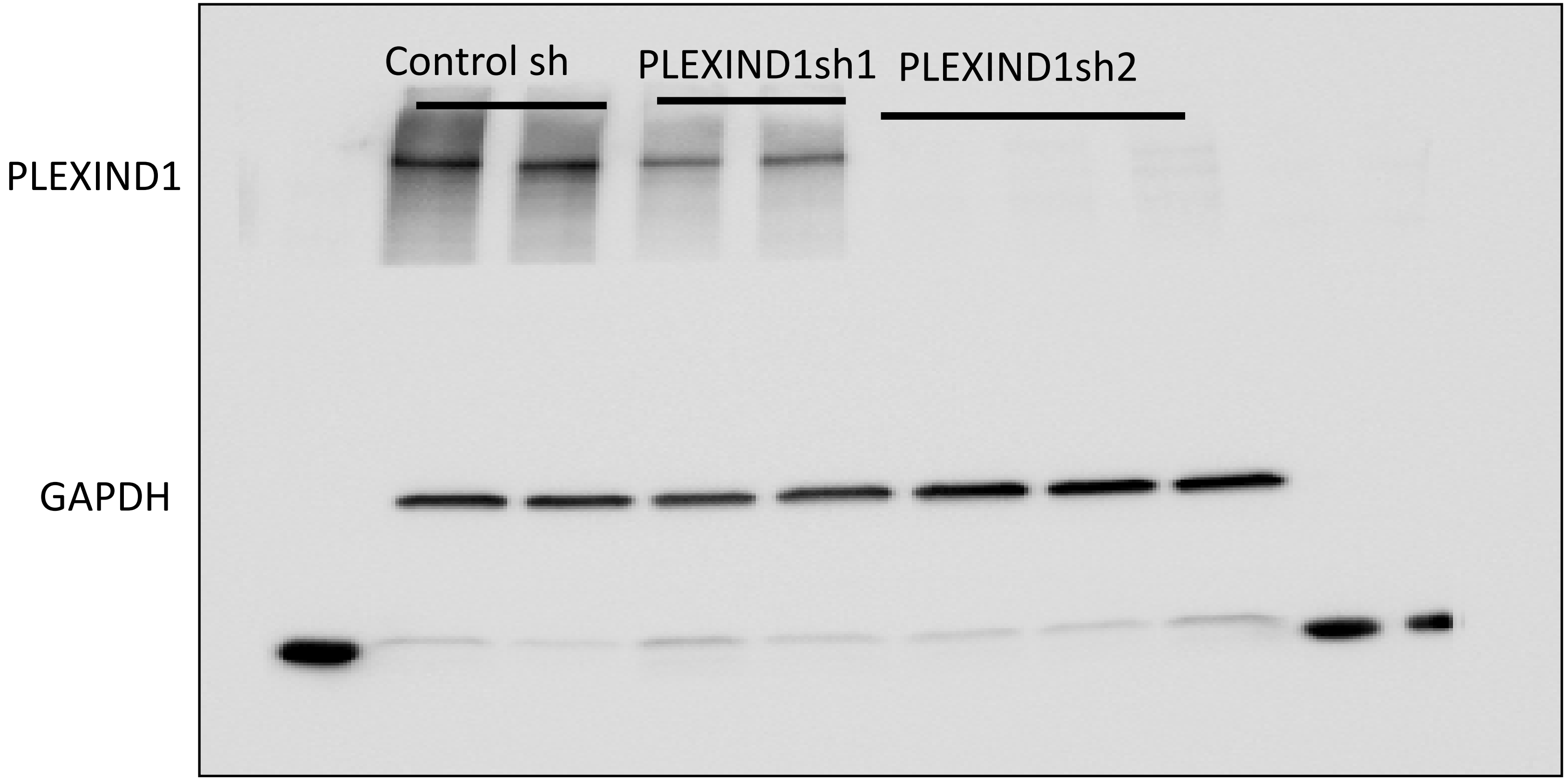

Figure: 3 Panel A Input

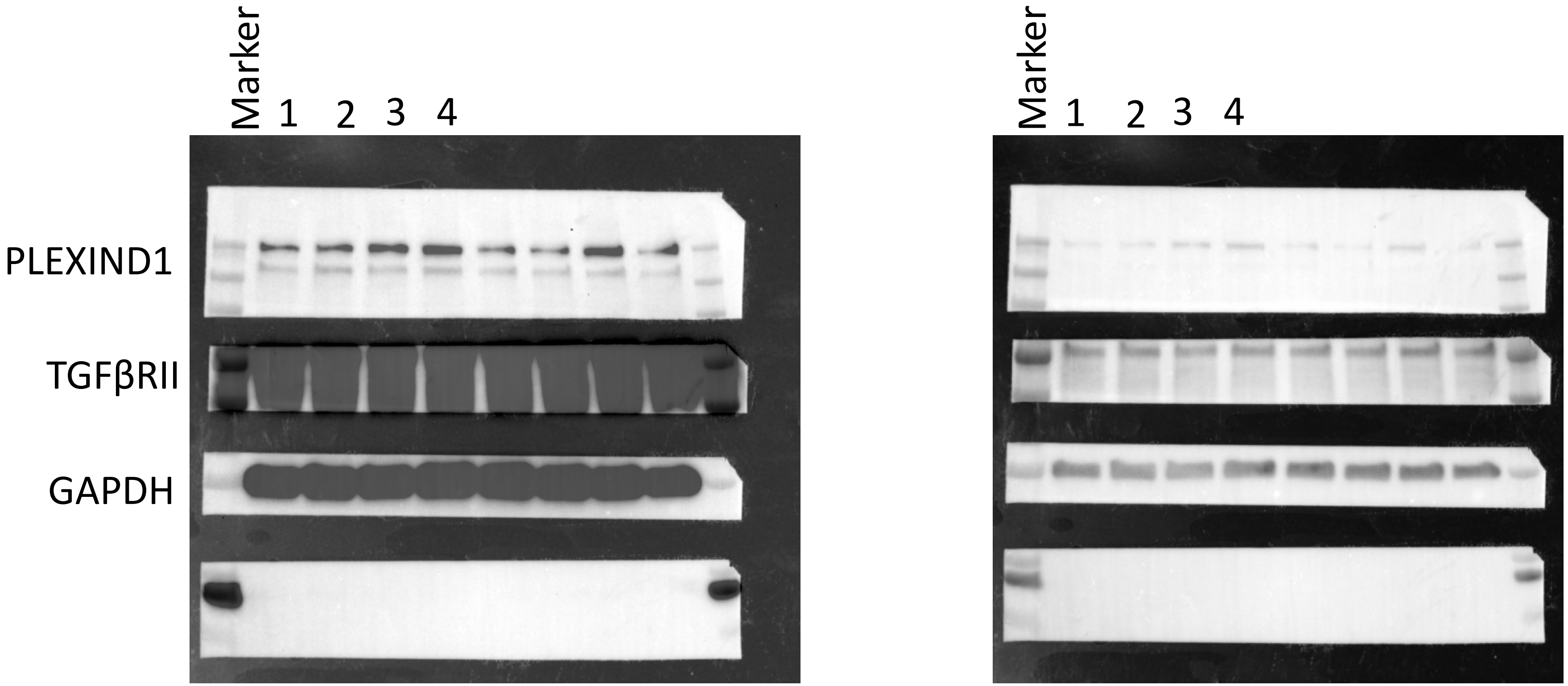

1: IgG

2:PANC-1 cells control

3:PANC-1 cells+10mins TGF-B induction

4:PANC-1 cells+30mins TGF-B induction

Figure: 3 Panel B IP: PLEXIND1

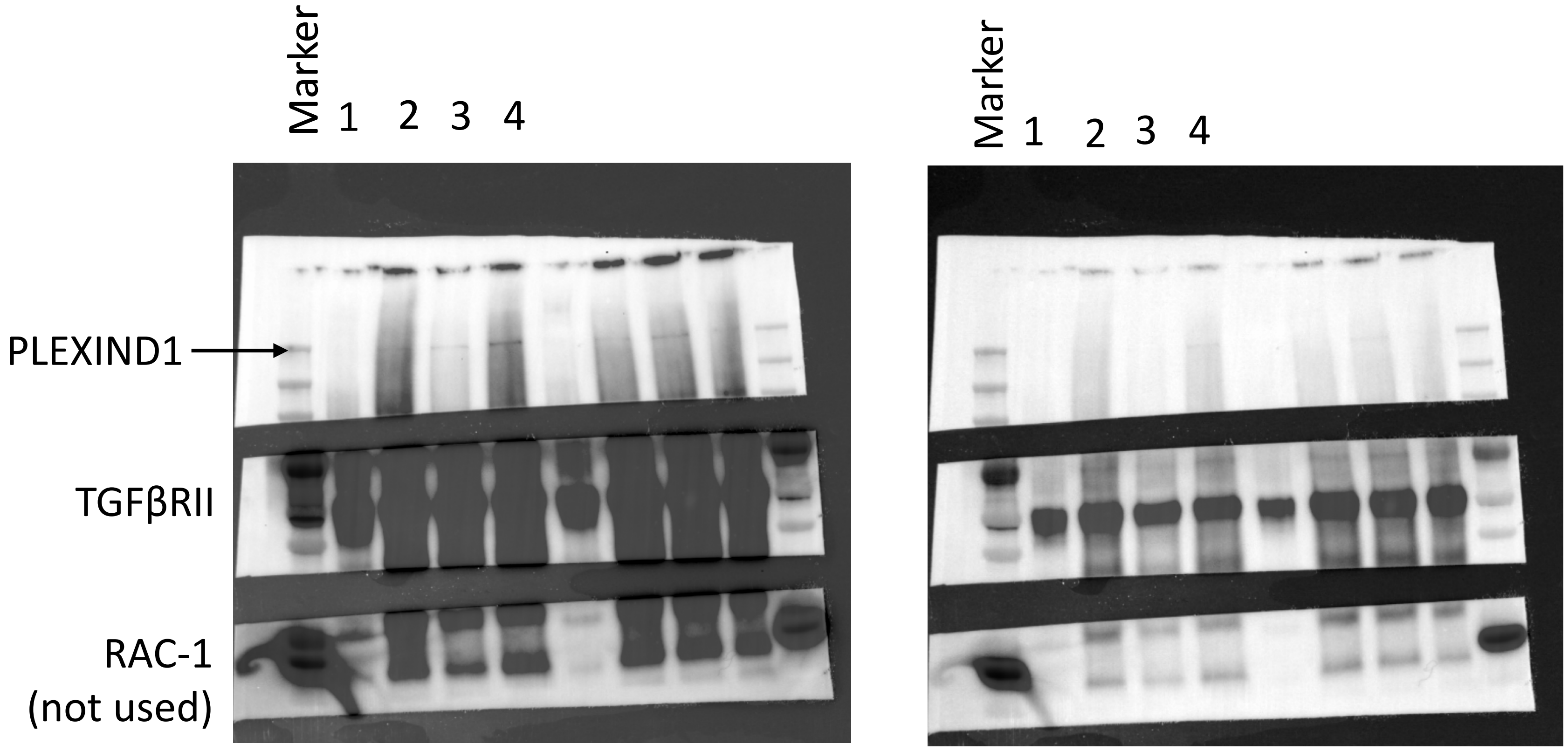

1: IgG
2:PANC-1 cells control
3:PANC-1 cells+10mins TGF-B induction
4:PANC-1 cells+30mins TGF-B induction

Figure: 3 Panel C IP: TGFBRII

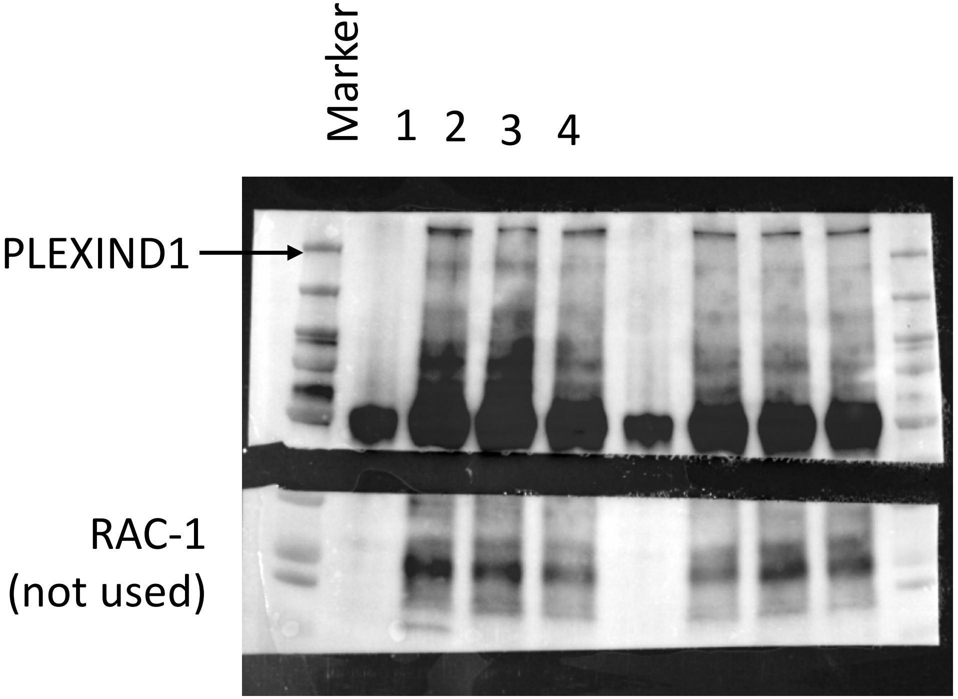

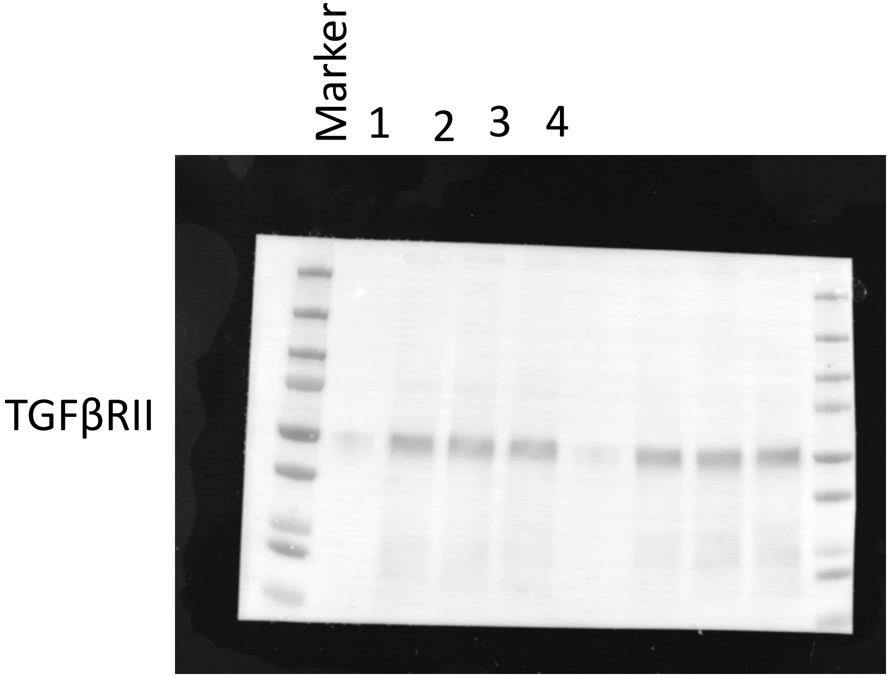

1: IgG
2:PANC-1 cells control
3:PANC-1 cells+10mins TGF-B induction
4:PANC-1 cells+30mins TGF-B induction

Figure: 4 Panel A

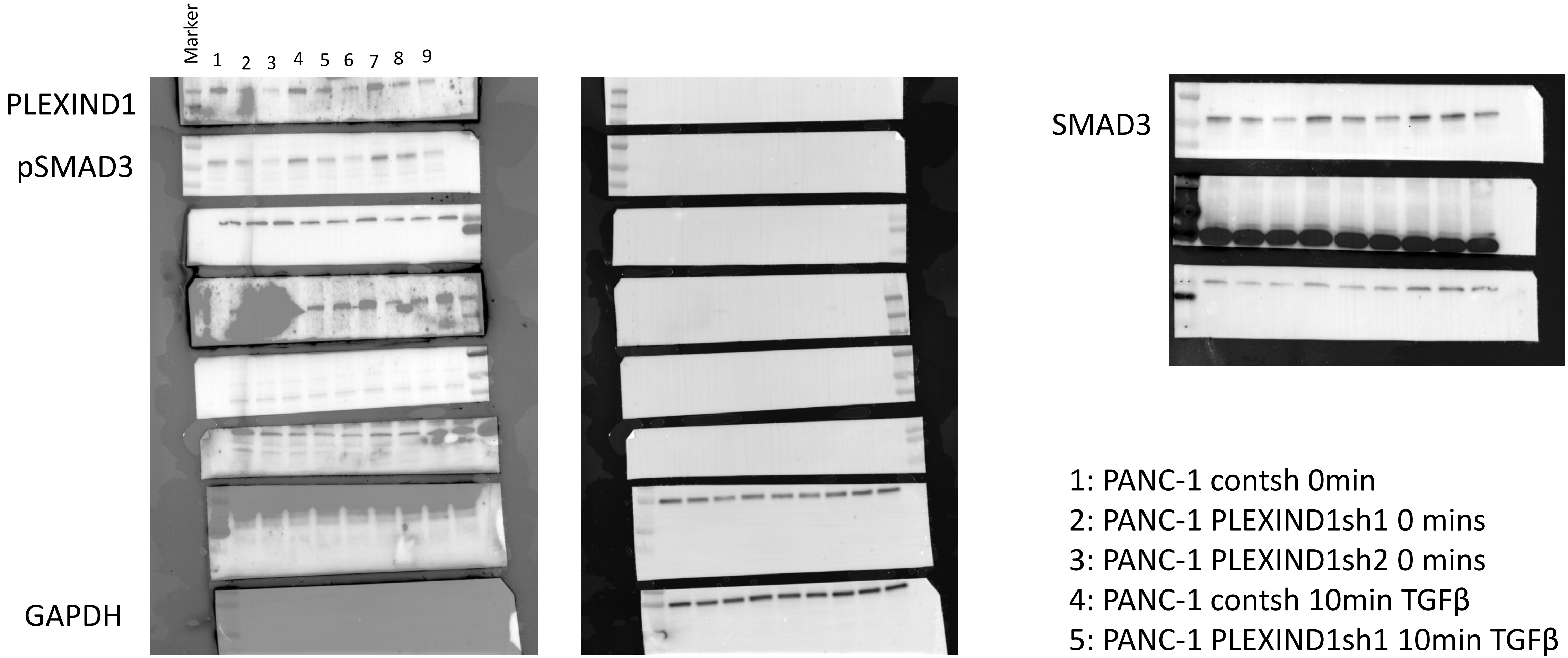

1: PANC-1 contsh 0min
2: PANC-1 PLEXIND1sh1 0 mins
3: PANC-1 PLEXIND1sh2 0 mins
4: PANC-1 contsh 10min TGFβ
5: PANC-1 PLEXIND1sh1 10min TGFβ
6: PANC-1 PLEXIND1sh2 10min TGFβ
7: PANC-1 contsh 30min TGFβ
8: PANC-1 PLEXIND1sh1 30min TGFβ
9: PANC-1 PLEXIND1sh2 30min TGFβ

Figure: 4 Panel B

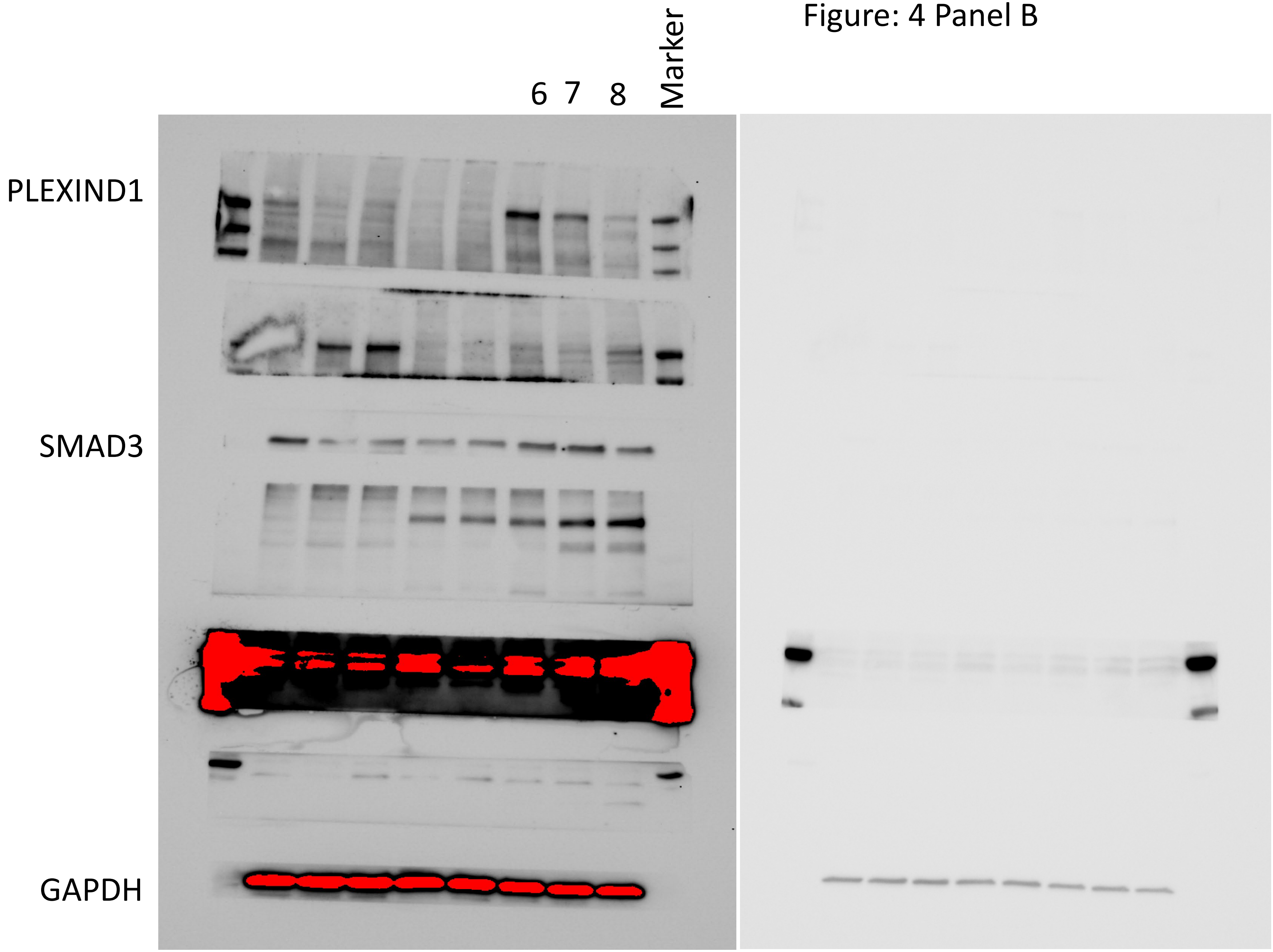

6: AsPC-1 cont sh
7: AsPC-1 PLEXIND1sh1
8: AsPC-1 PLEXIND1sh2

Figure: 4 Panel C

4535

5 6 7

PLEXIND1

pSMAD3

SMAD3

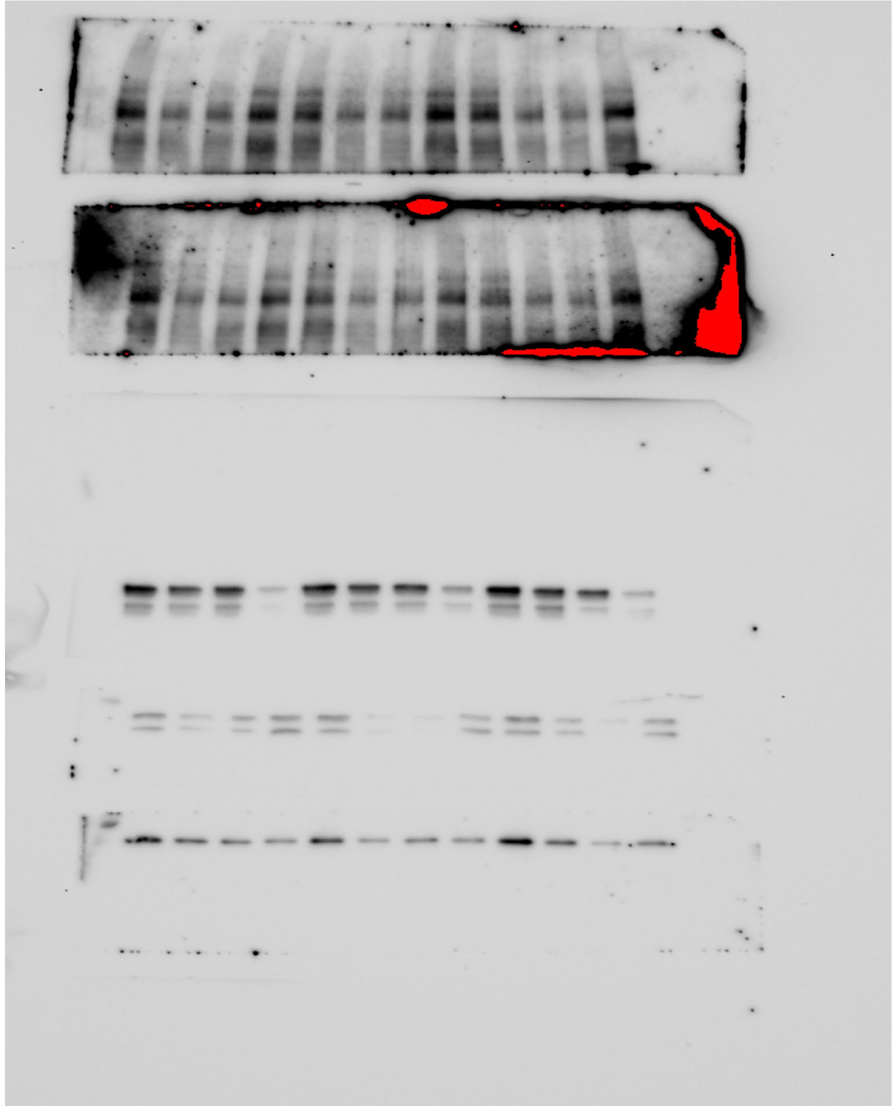

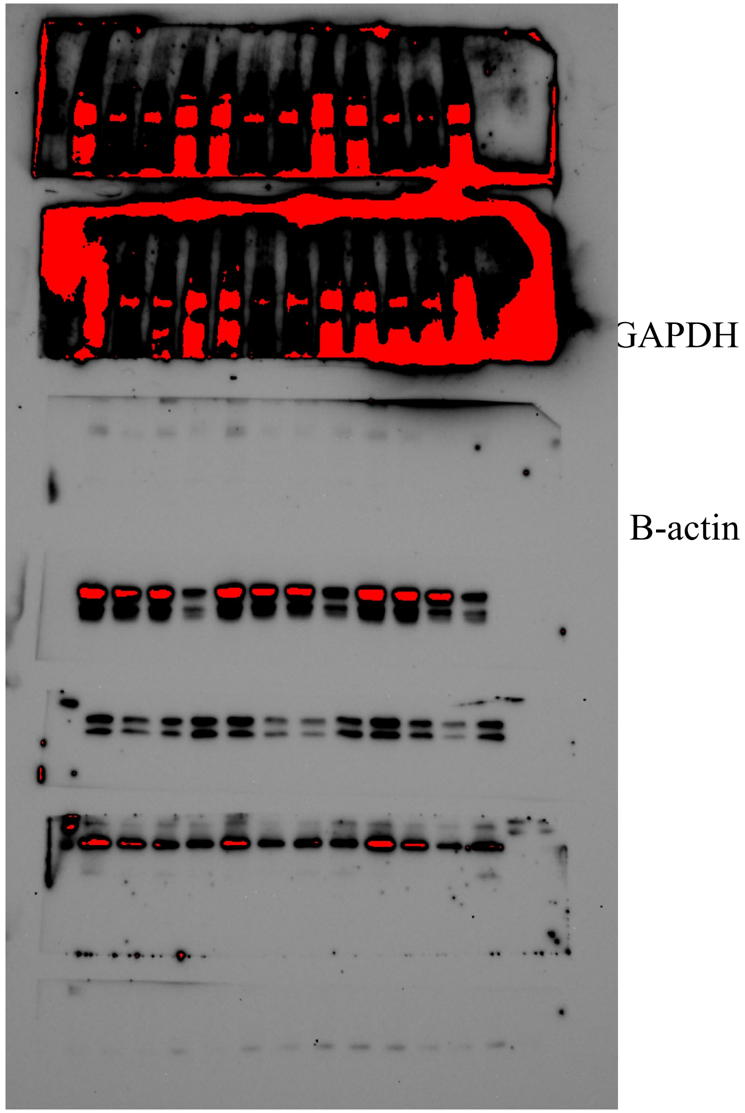

4535

5 6 7

GAPDH

B-actin

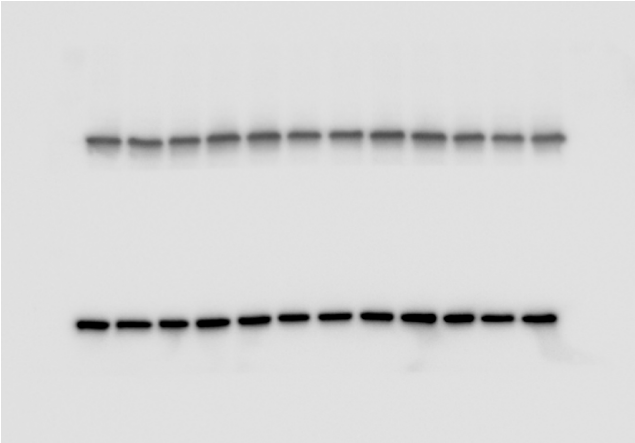

5: 4535 cont sh
6: 4535 PLEXIND1sh1
7: 4535 PLEXIND1sh2

Figure: 4 Panel D

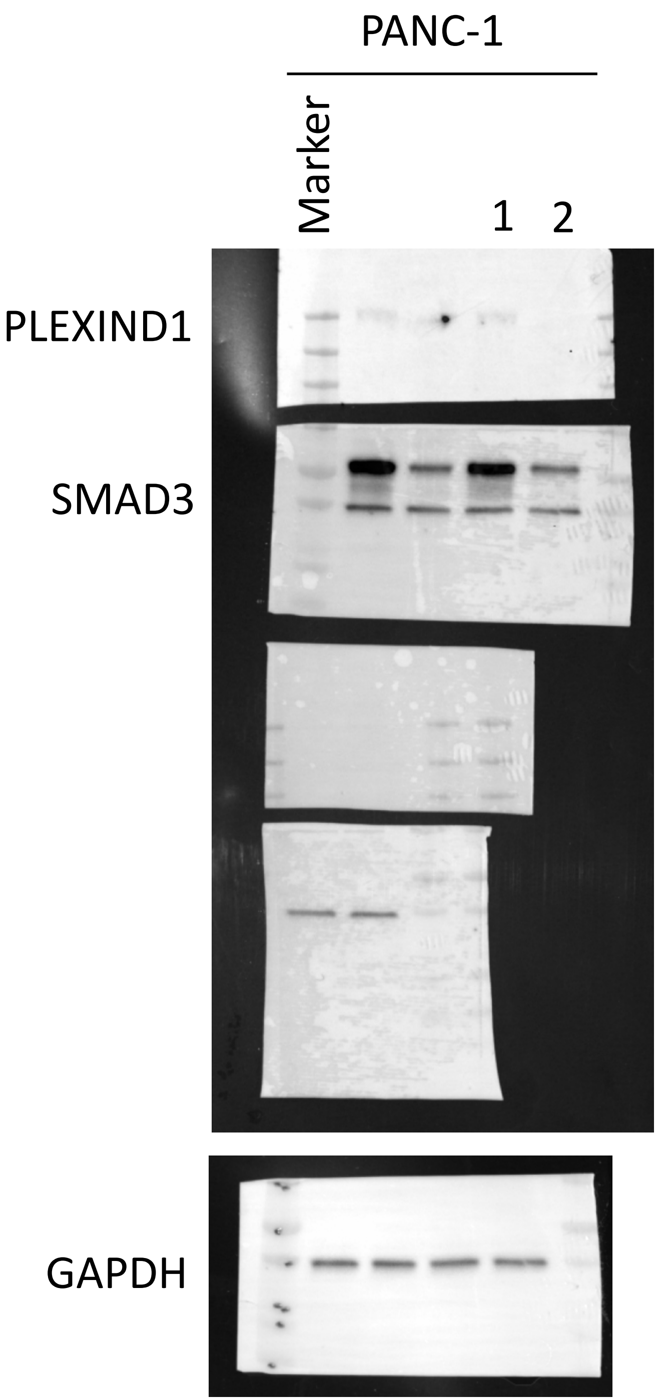

Data for SMAD3 sh2 on slide 13

1: PANC-1 cont sh
2:PANC-1 SMAD3sh1

Figure: 4 Panel E

1 2

PLEXIND1

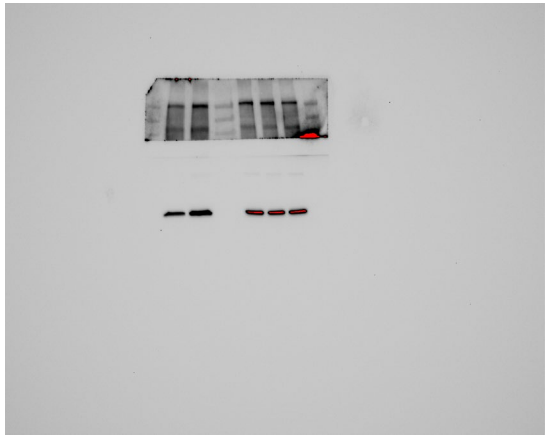

GAPDH

1 2

SMAD3

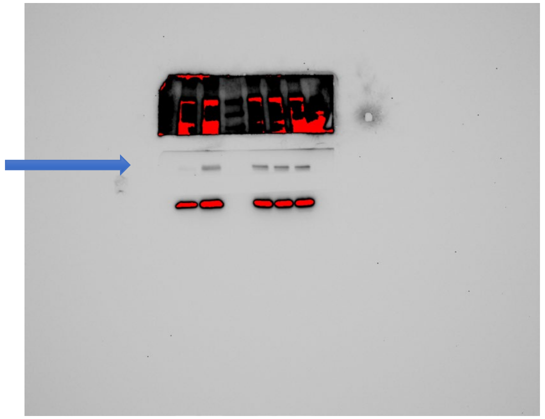

Beta-actin

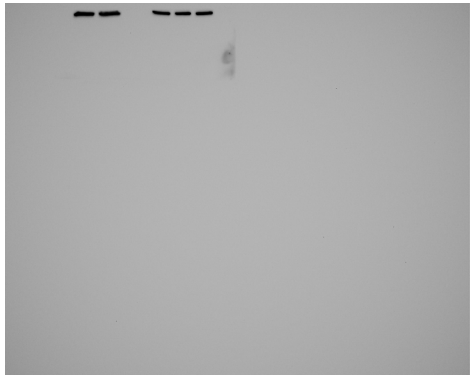

AsPC-1

PLEXIND1

SMAD3

Beta-actin

GAPDH

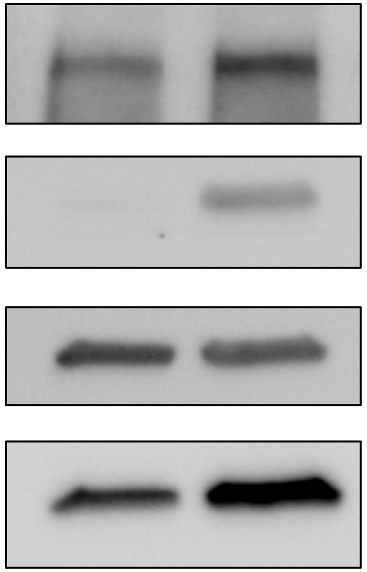

SMAD3 sh	+	-
Control sh	-	+

1: PANC-1 SMAD3sh1

2:PANC-1 cont sh

Figure: 4 Panel D and E

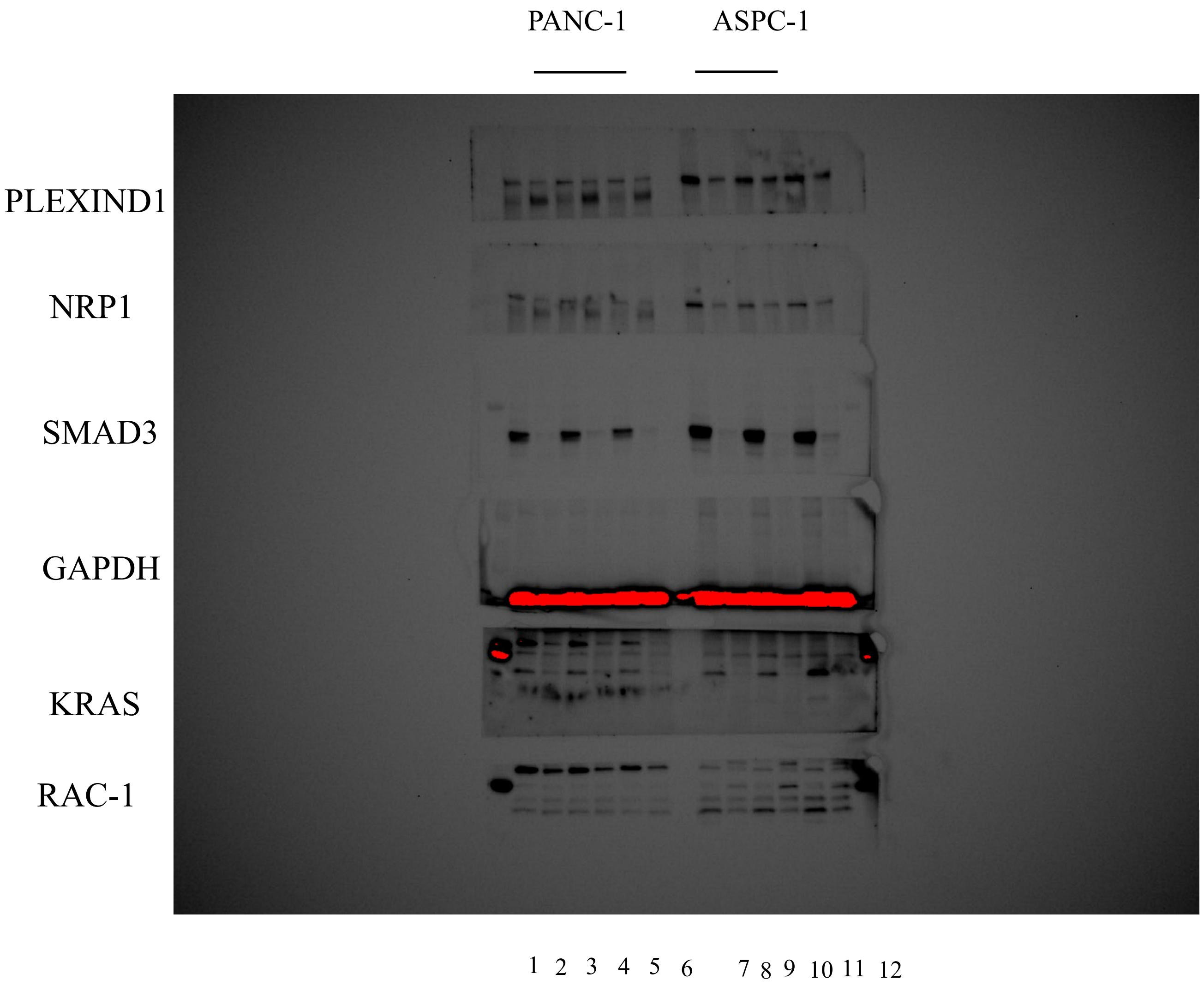

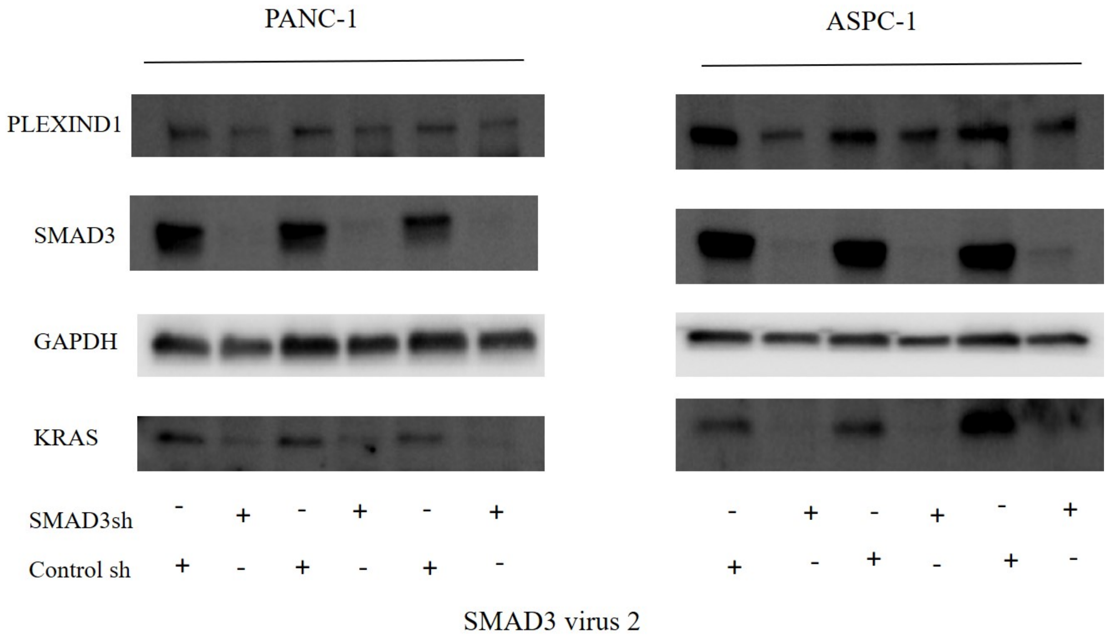

Samples:
1,3,5: PANC-1 cont sh
2,4,6:PANC-1 SMAD3 sh2
7,9,11: AsPC-1 cont sh
8,10,12: AsPC-1 SMAD3 sh2

Figure: 4 Panel H

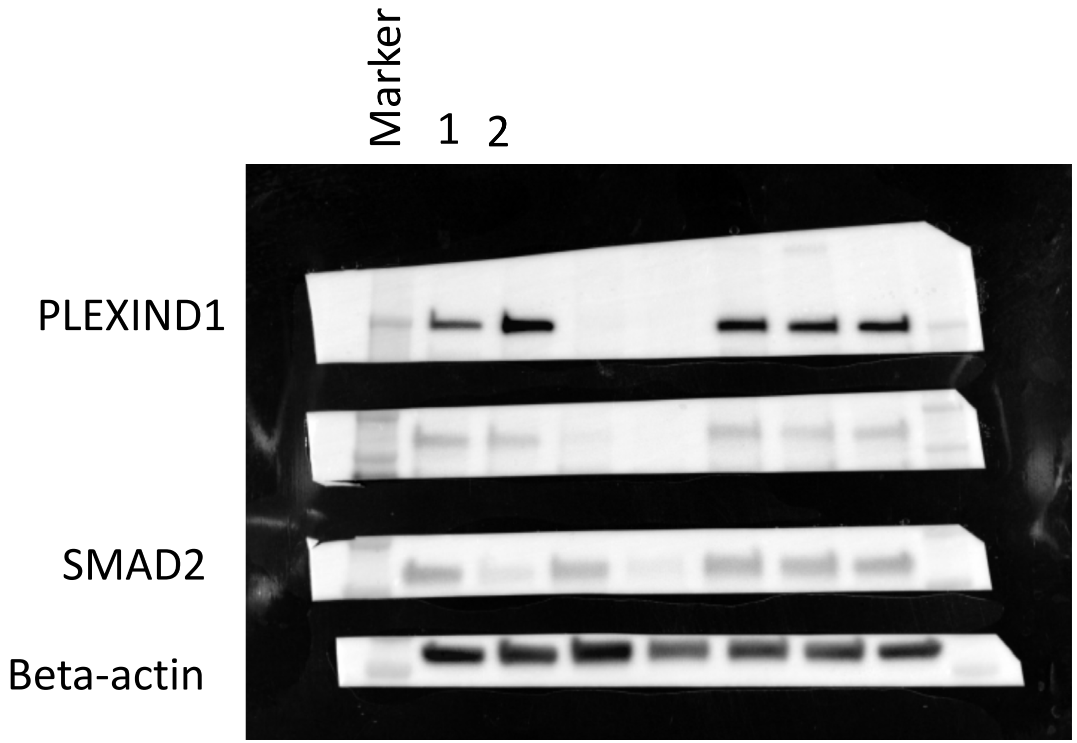

1: PANC-1 cont sh
2:PANC-1 SMAD2 sh1

Figure: 5 Panel C

PLEXIND1

RAC-1

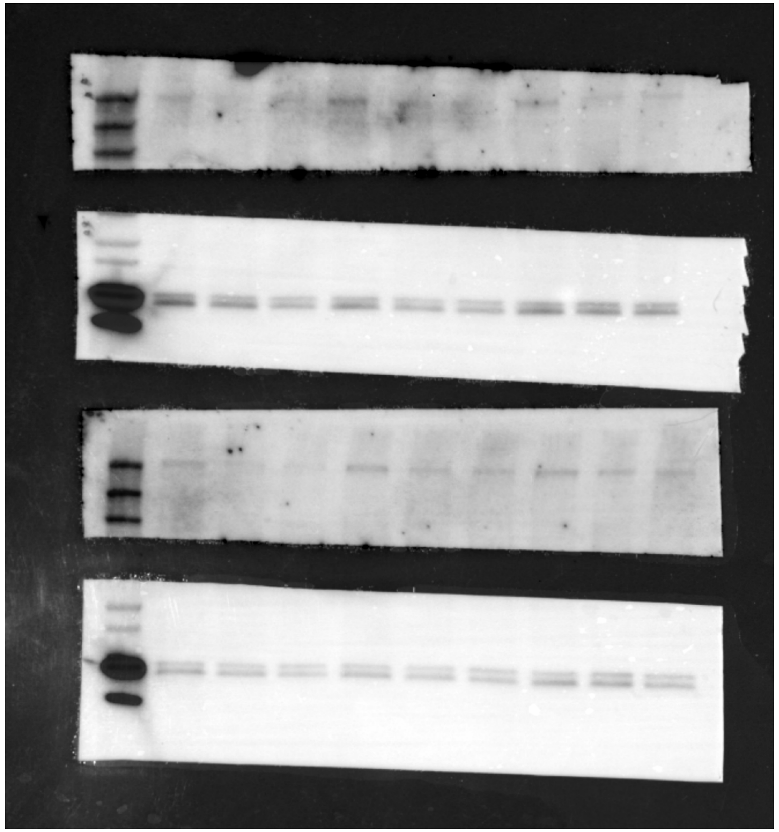

Marker 1 2 3

GAPDH

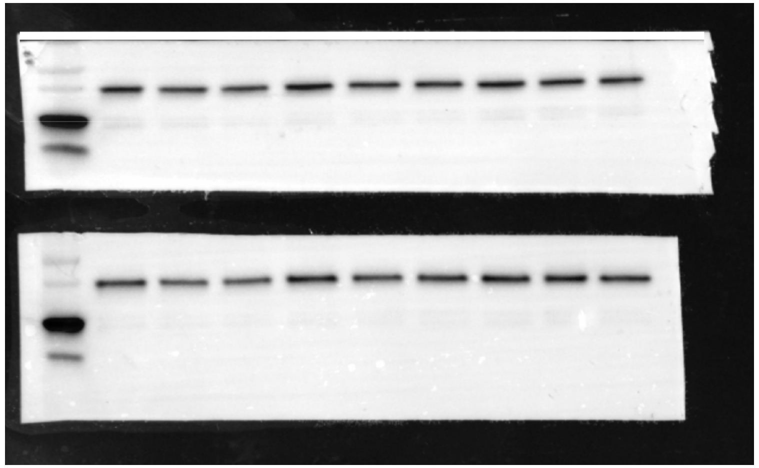

1: PANC-1 cont sh
2: PANC-1 PLEXIND1sh1
3: PANC-1 PLEXIND1sh2

Figure: 5 Panel D

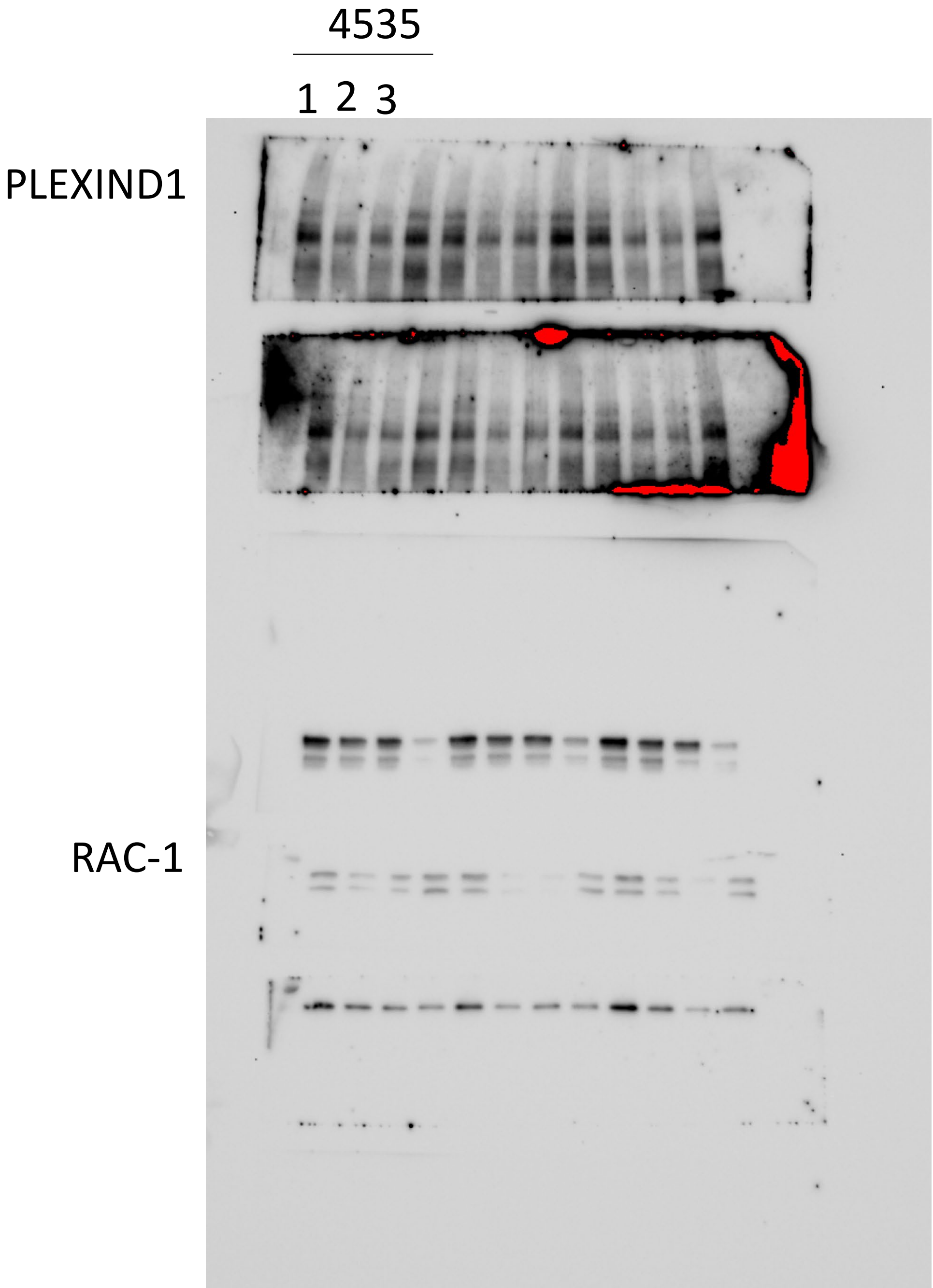

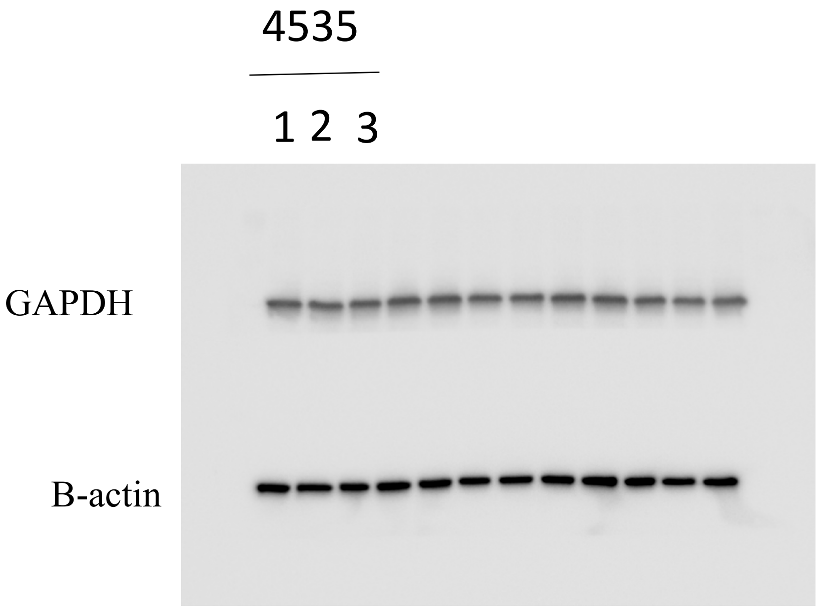

1: 4535 cont sh
2: 4535 PLEXIND1sh1
3: 4535 PLEXIND1sh2

Figure: 5 Panel E

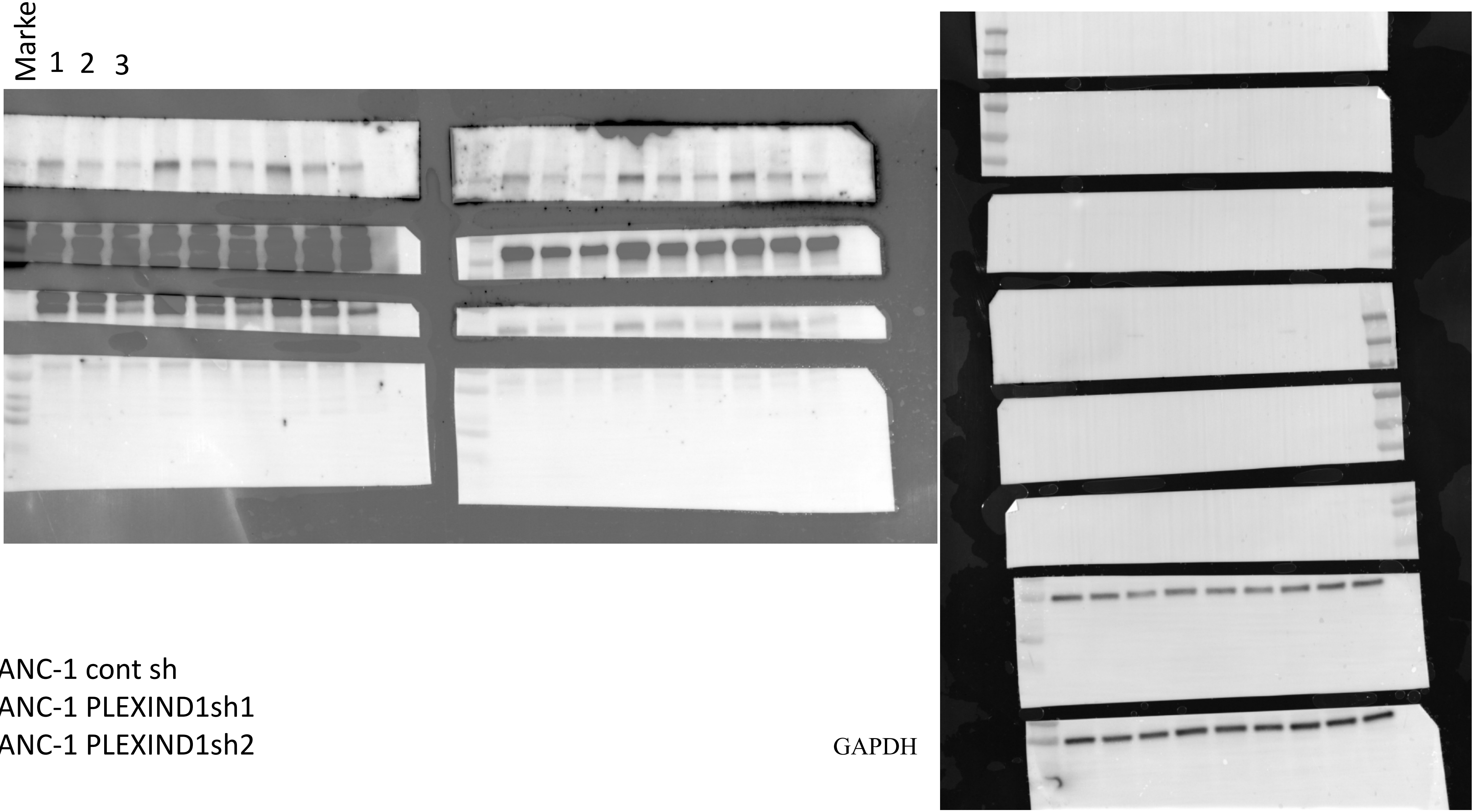

1: PANC-1 cont sh
2: PANC-1 PLEXIND1sh1
3: PANC-1 PLEXIND1sh2

Figure: 5 Panel E

Marker 1 2 3

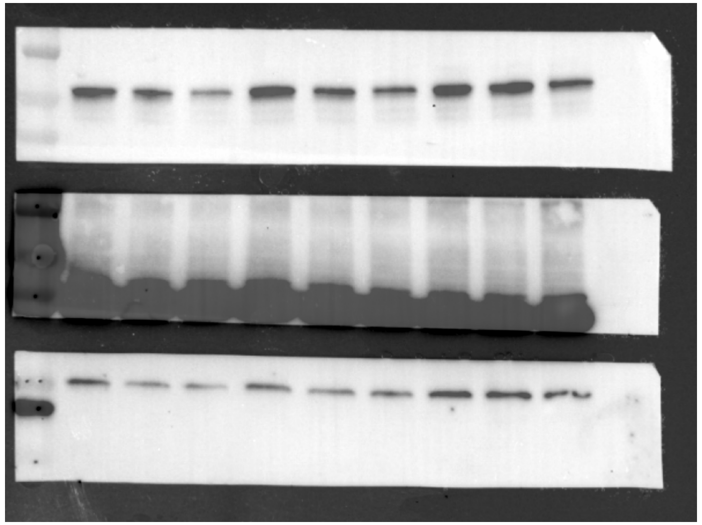

KRAS

Marker 1 2 3

GAPDH

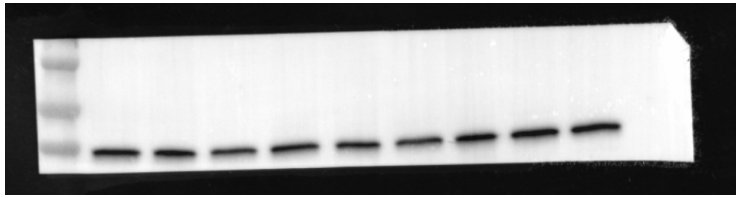

1: PANC-1 cont sh
2: PANC-1 PLEXIND1sh1
3: PANC-1 PLEXIND1sh2

Figure: 5 Panel F

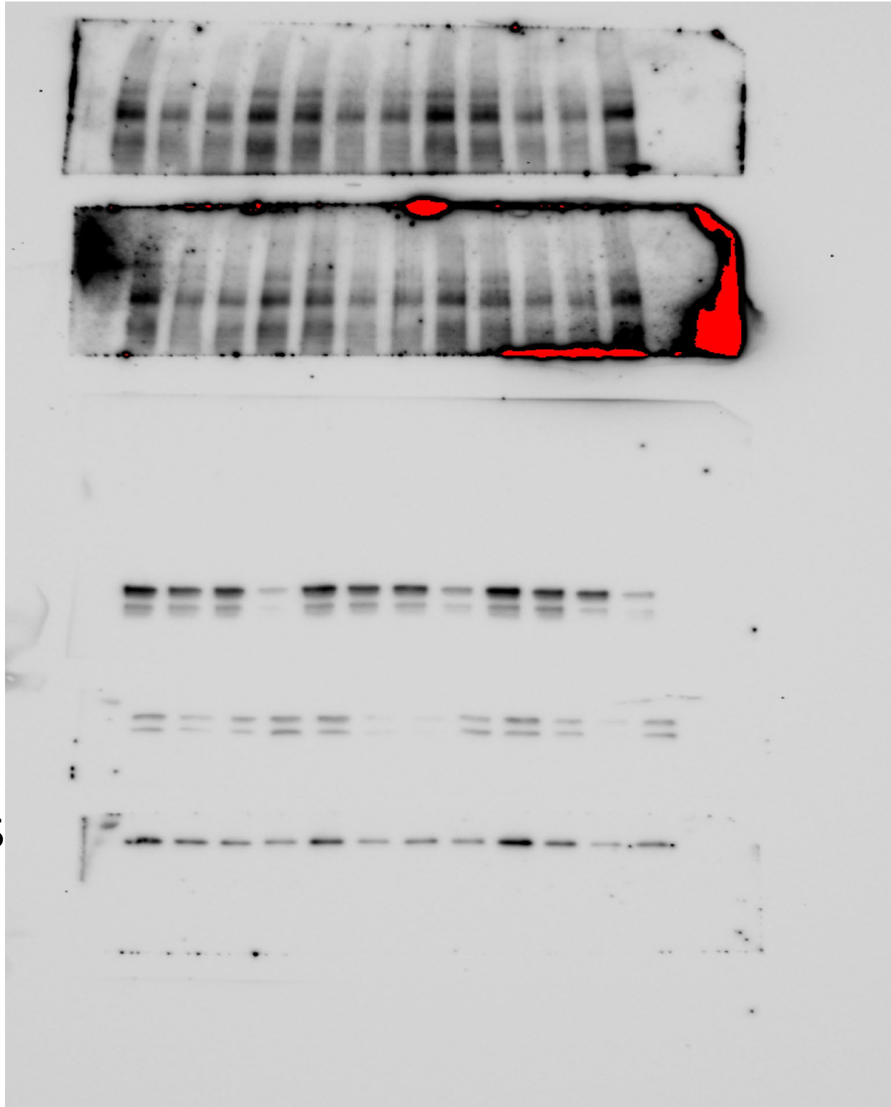

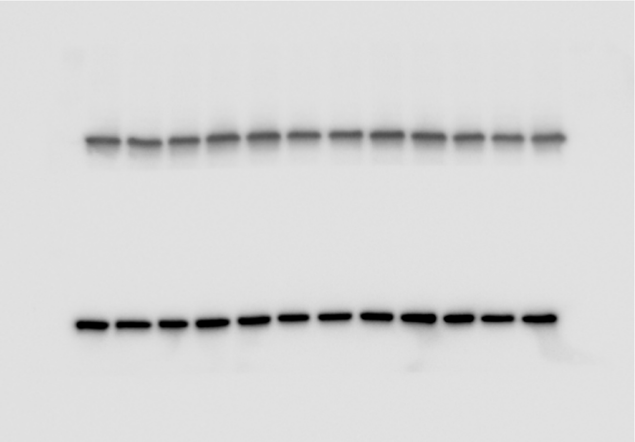

5: 4535 cont sh
6: 4535 PLEXIND1sh1
7: 4535 PLEXIND1sh2

Figure: 6 Panel B

Marker 3 4

PLEXIND1

GAPDH

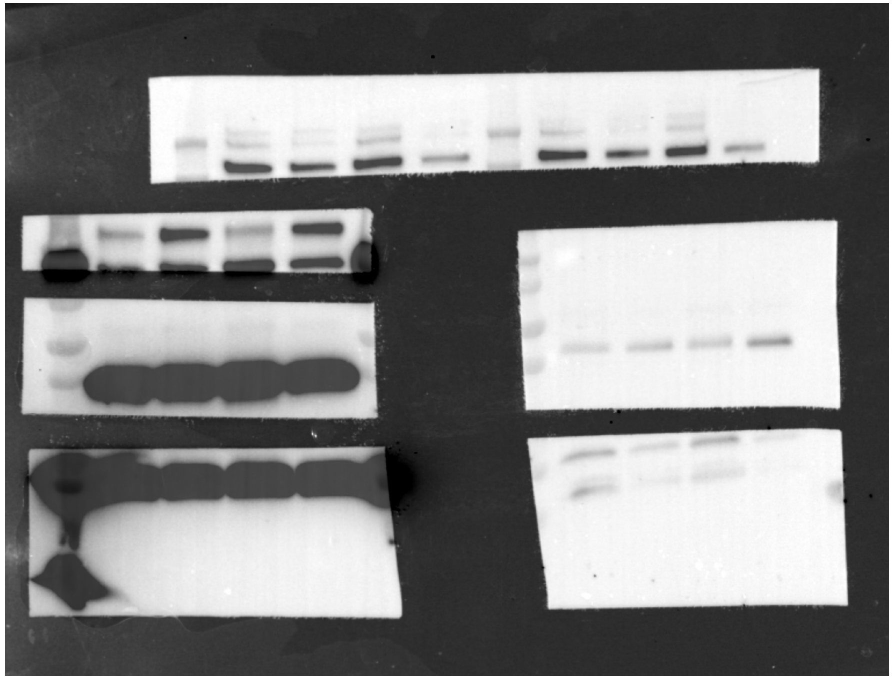

Marker 3 4

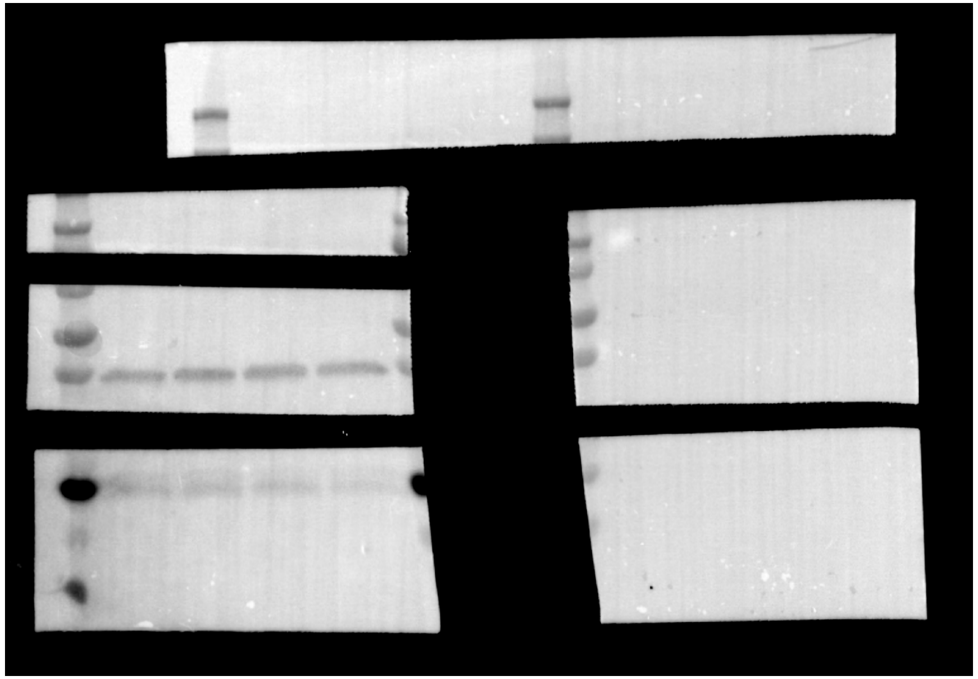

3: BxPC-3 cont sh

4: BxPC-3 PLEXIND1sh1

Figure: 7 Panel F

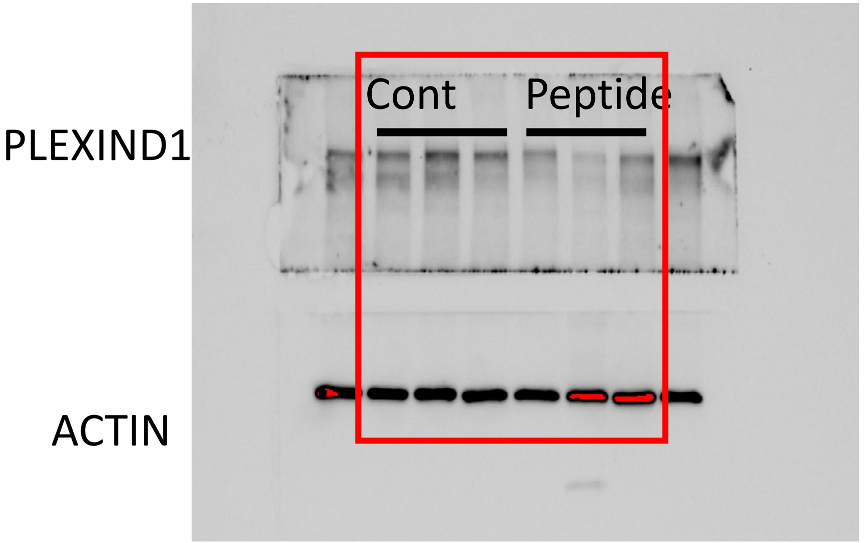

Supplementary Fig 1 Panel A

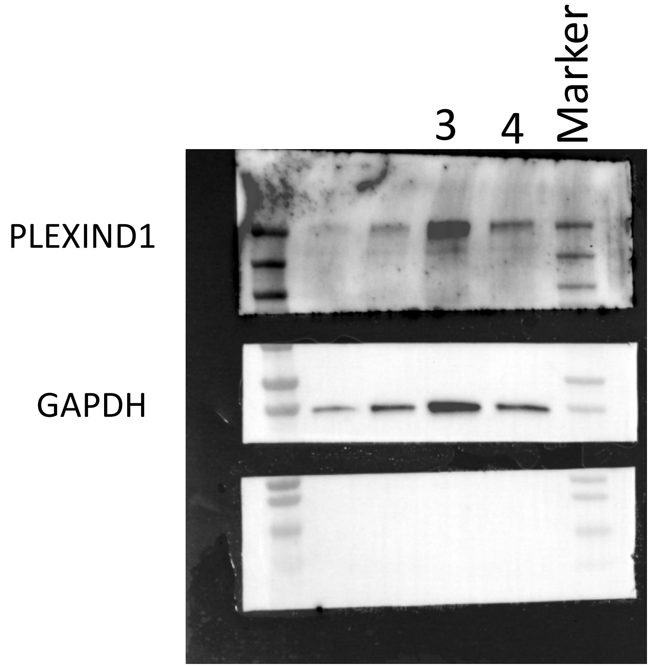

PANC-1 sgRNA system
3: PANC-1 Cas9 control
4: PANC-1 Cas9 +Dox

Supplementary Fig 1 Panel D

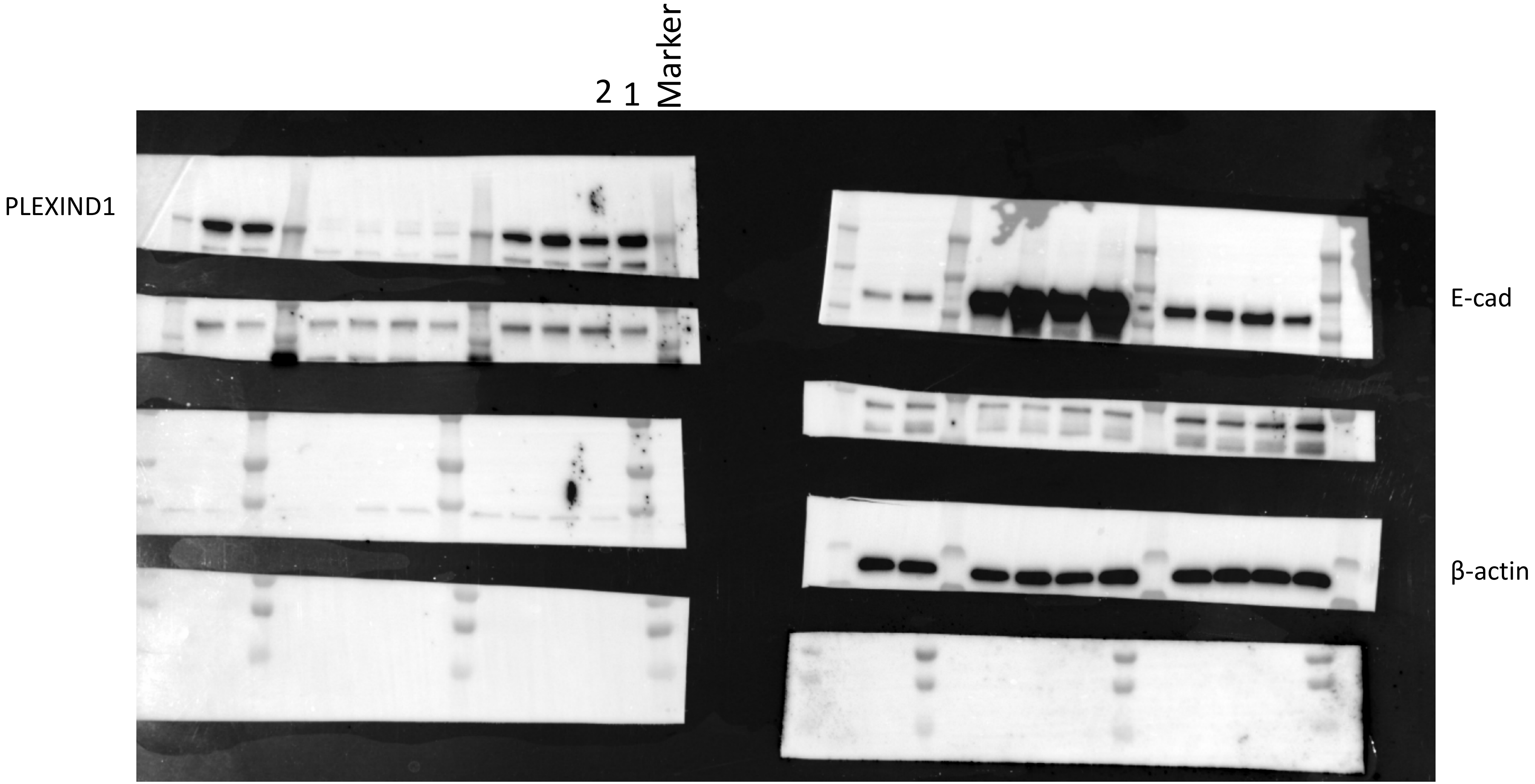

1: PANC-1 cont sh
2: PANC-1 PLEXIND1sh1

Supplementary Fig 2 Panel A

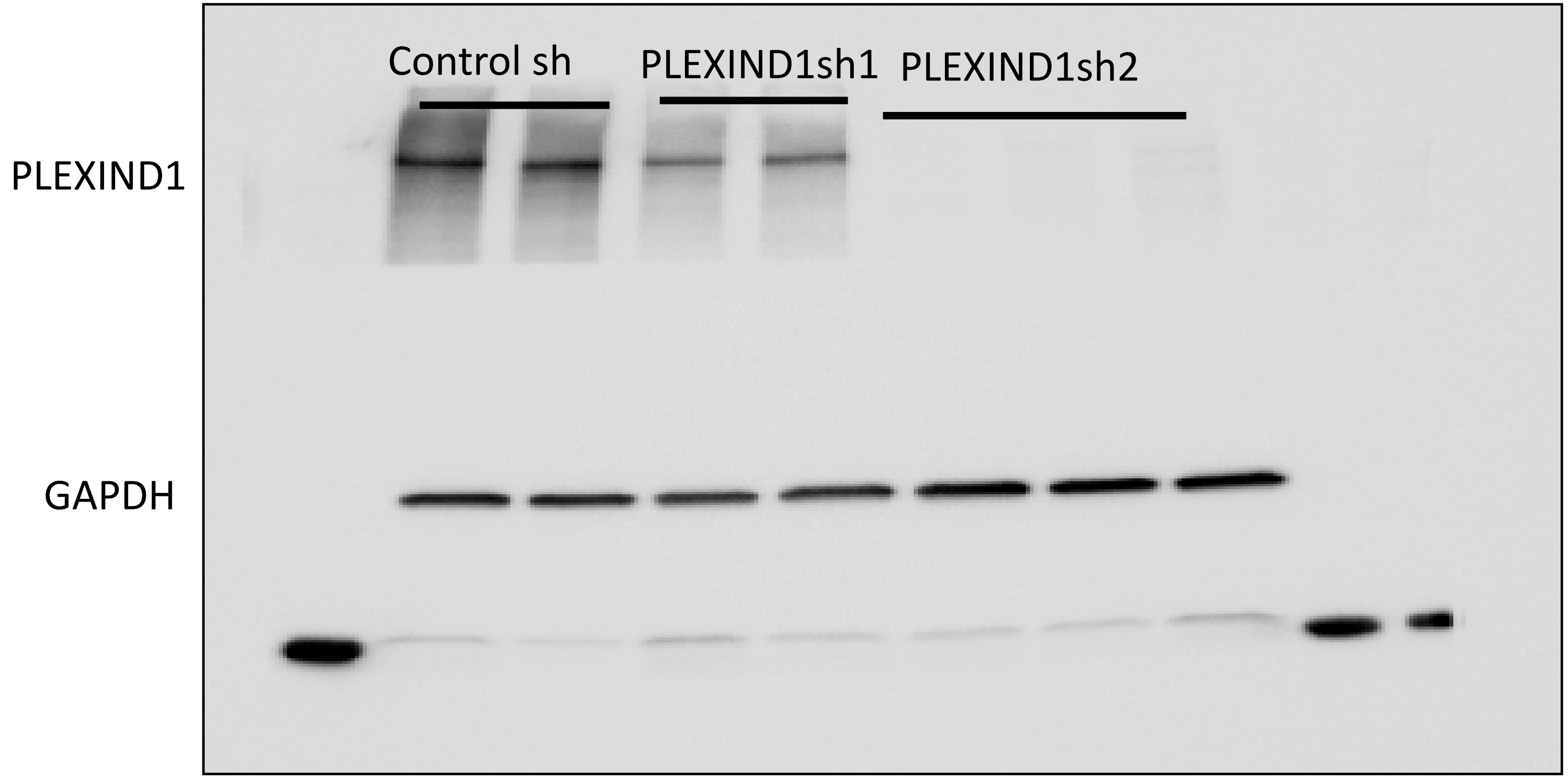

Supplementary Fig 3 Panel A

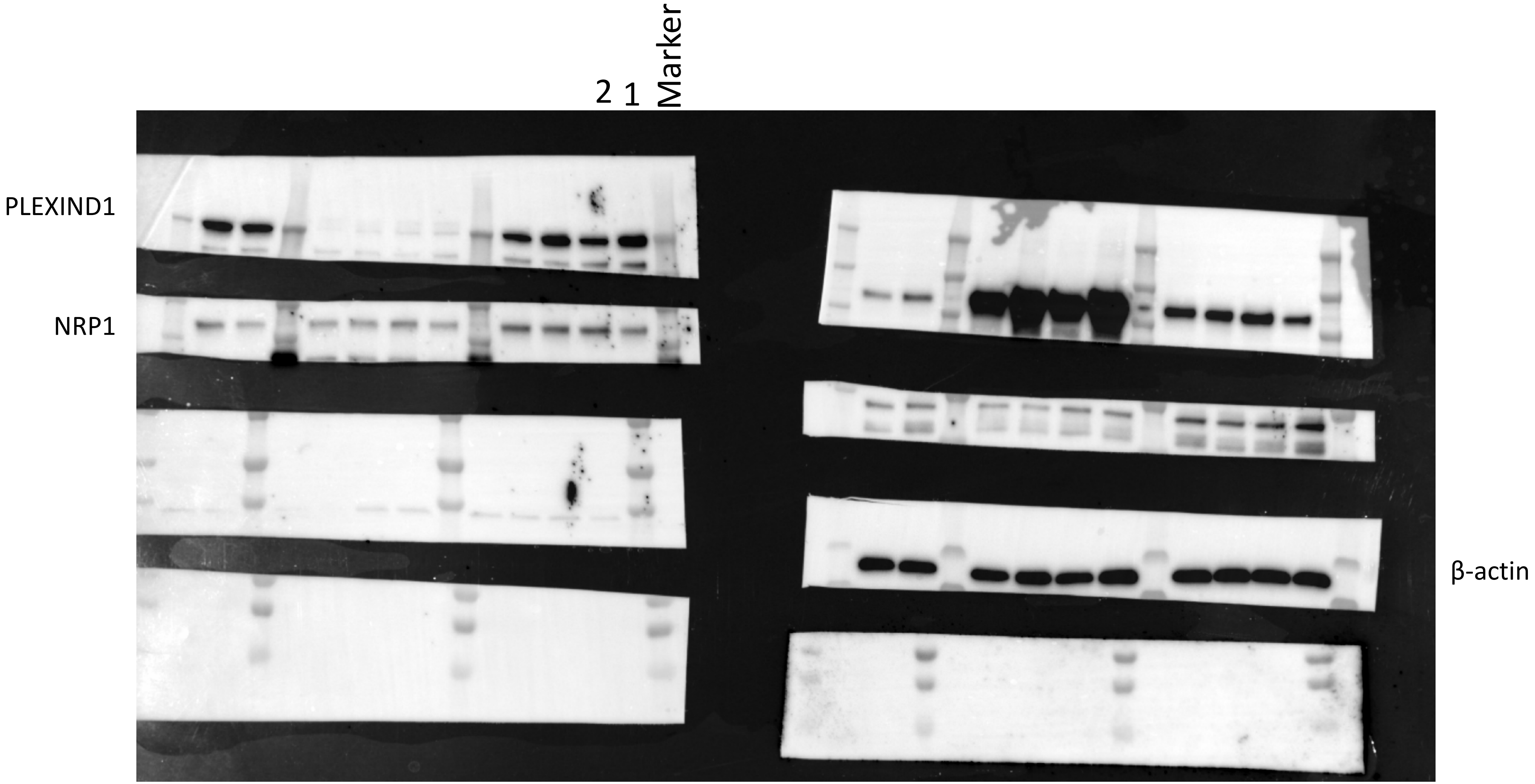

1: PANC-1 cont sh
2: PANC-1 PLEXIND1sh1

Supplementary Fig 3 Panel B

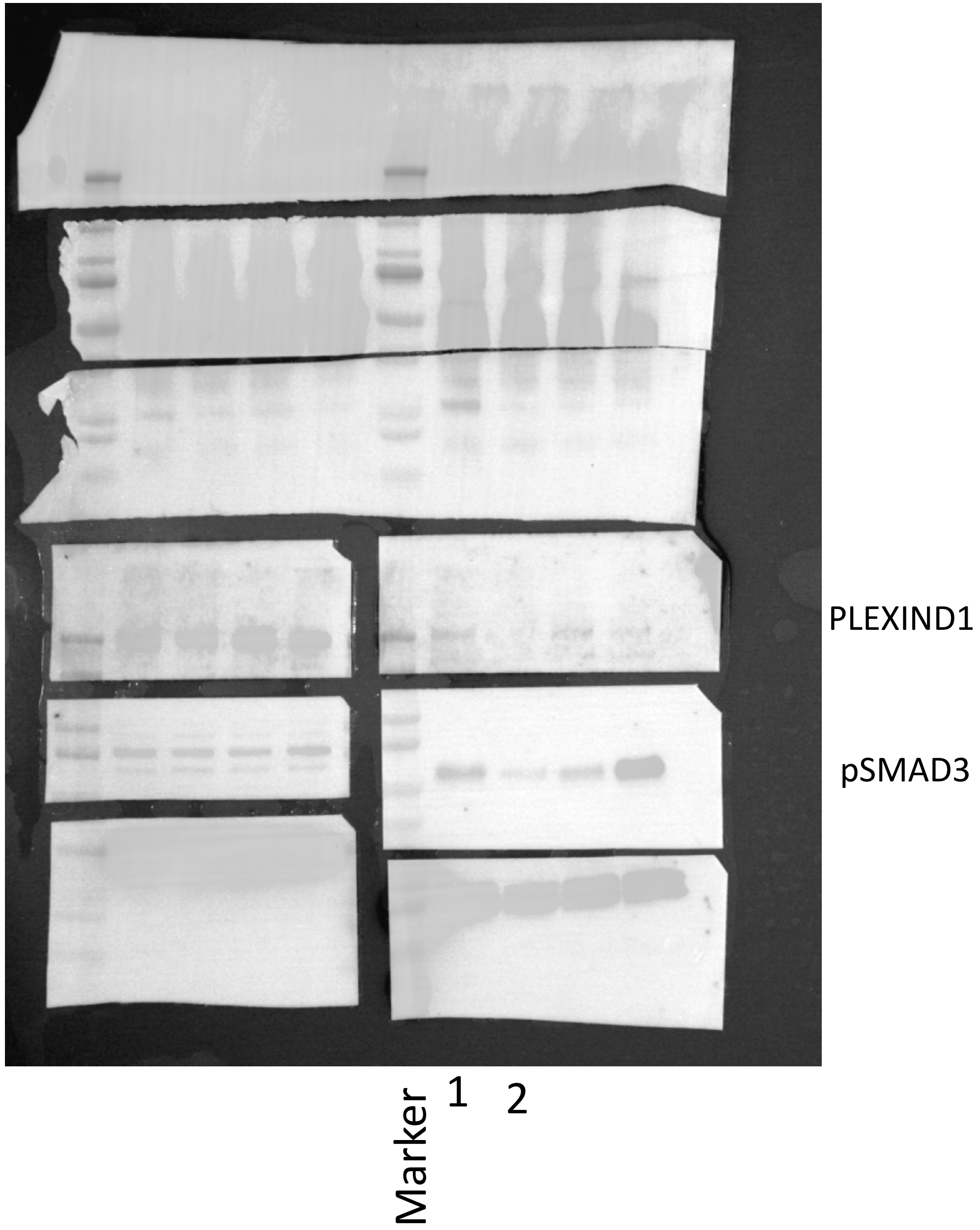

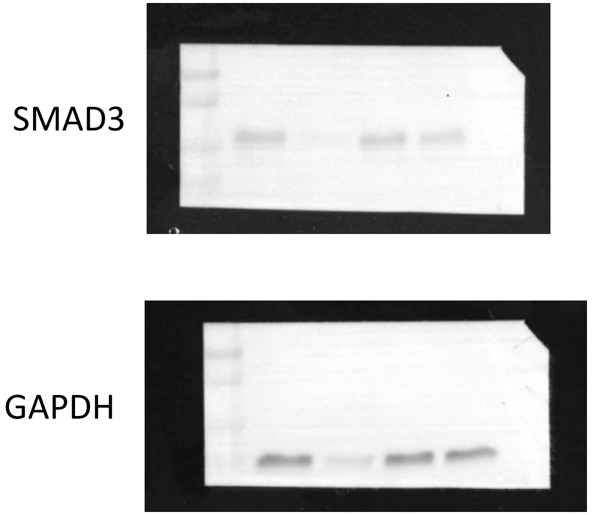

PANC-1 sgRNA system
1: PANC-1 Cas9 control
2: PANC-1 Cas9 +Dox

Supplementary Fig 3 Panel C

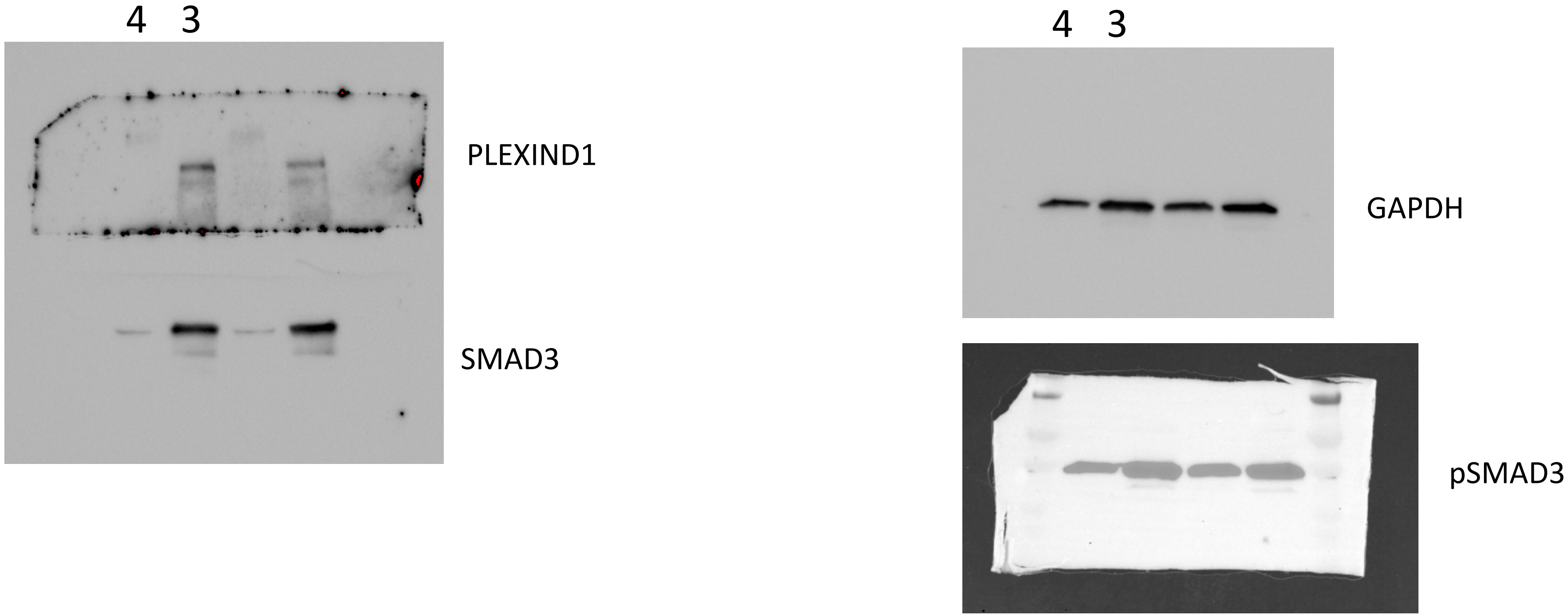

AsPC-1 sgRNA system
3: AsPC-1 Cas9 control
4: AsPC-1 Cas9 +Dox

Supplementary Fig 3 Panel D

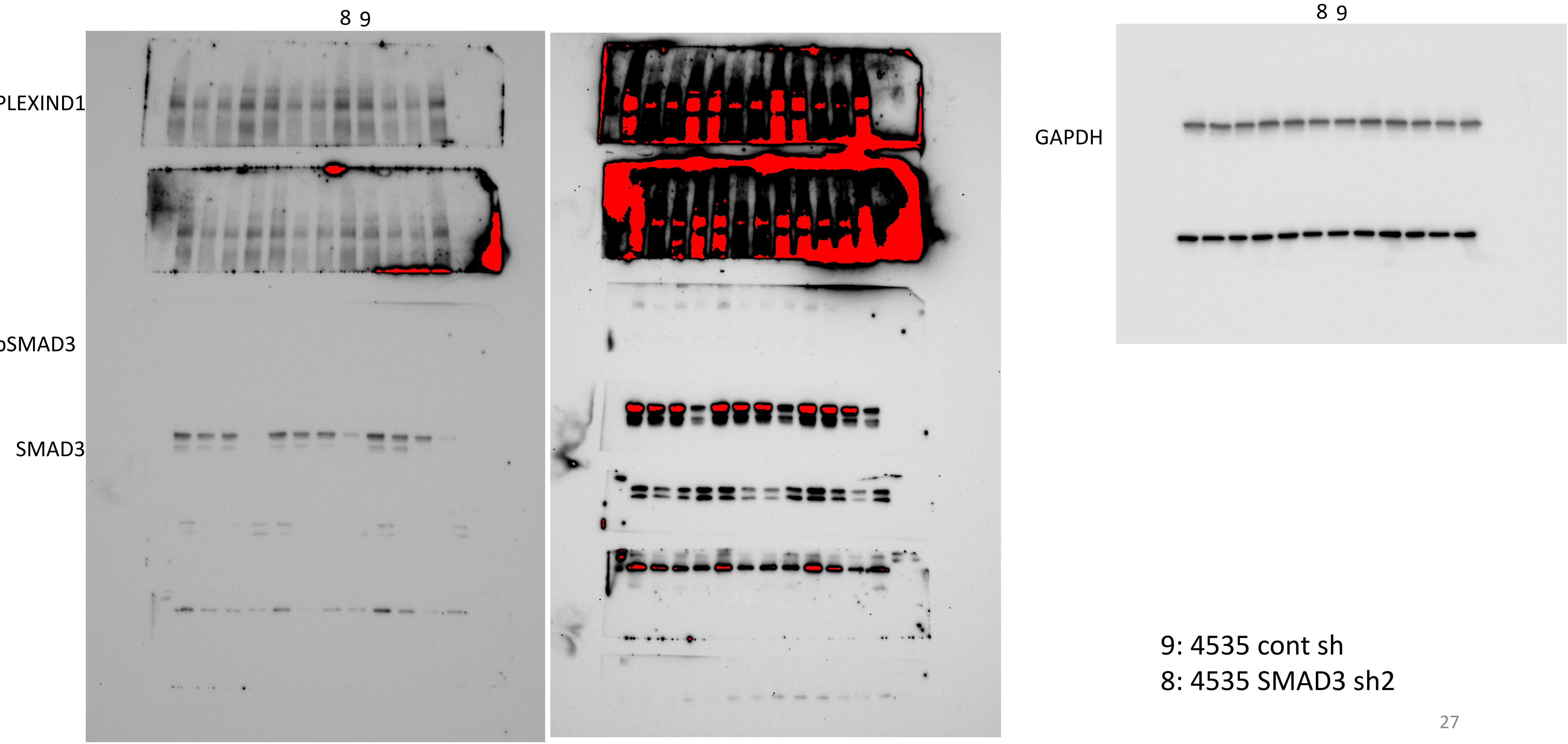

9: 4535 cont sh
8: 4535 SMAD3 sh2

Supplementary Fig 3 Panel F

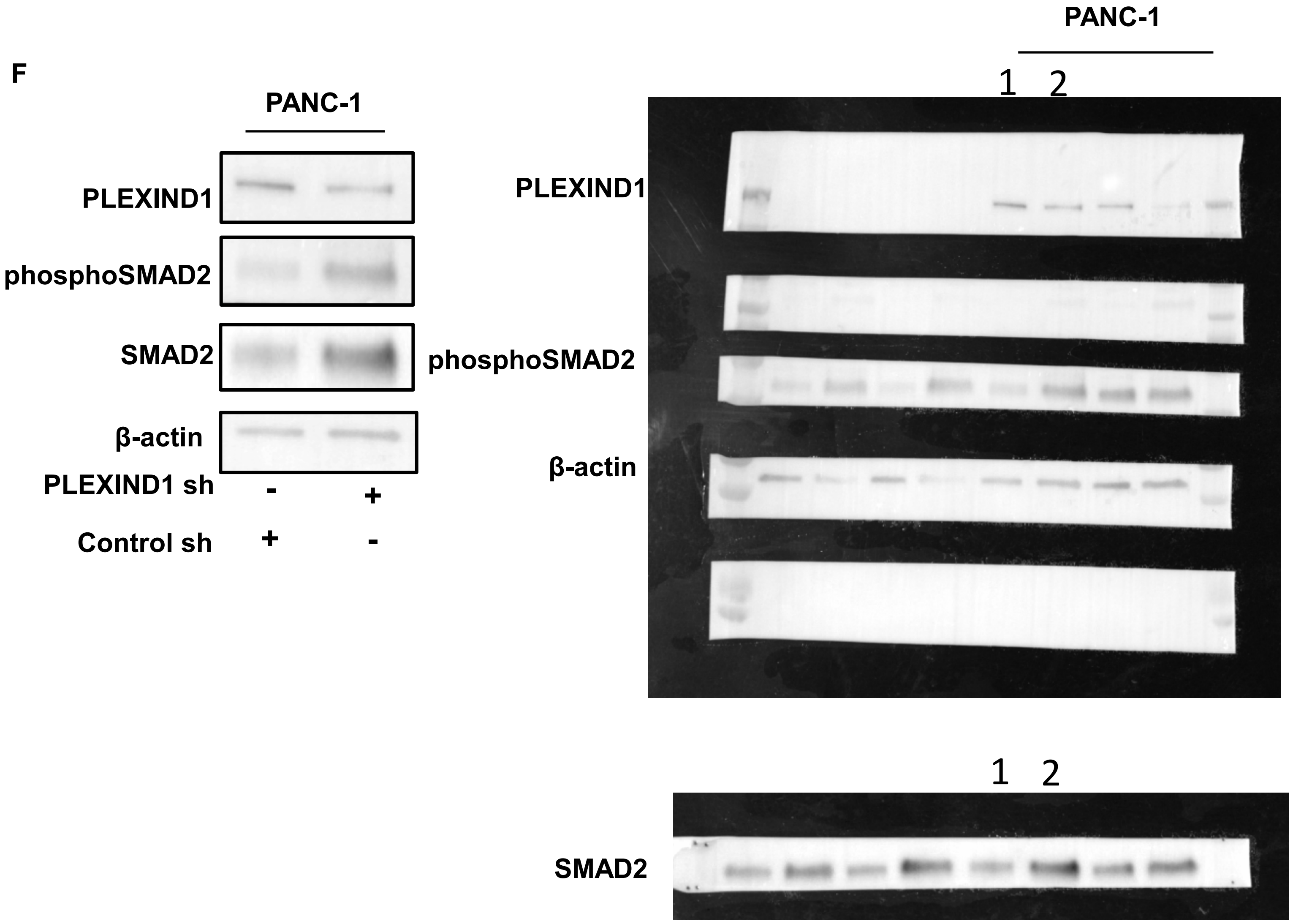

1: PANC-1 cont sh
2:PANC-1 PLEXIND1sh1

Supplementary Fig 4 Panel A

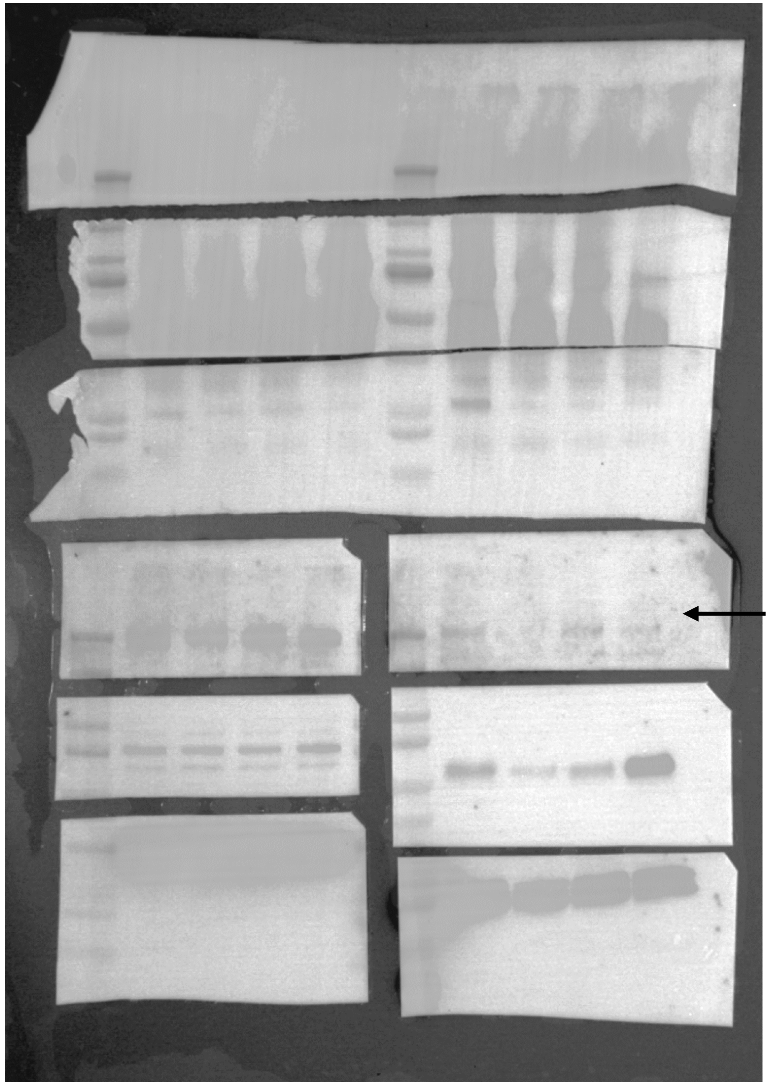

PLEXIND1

1 2

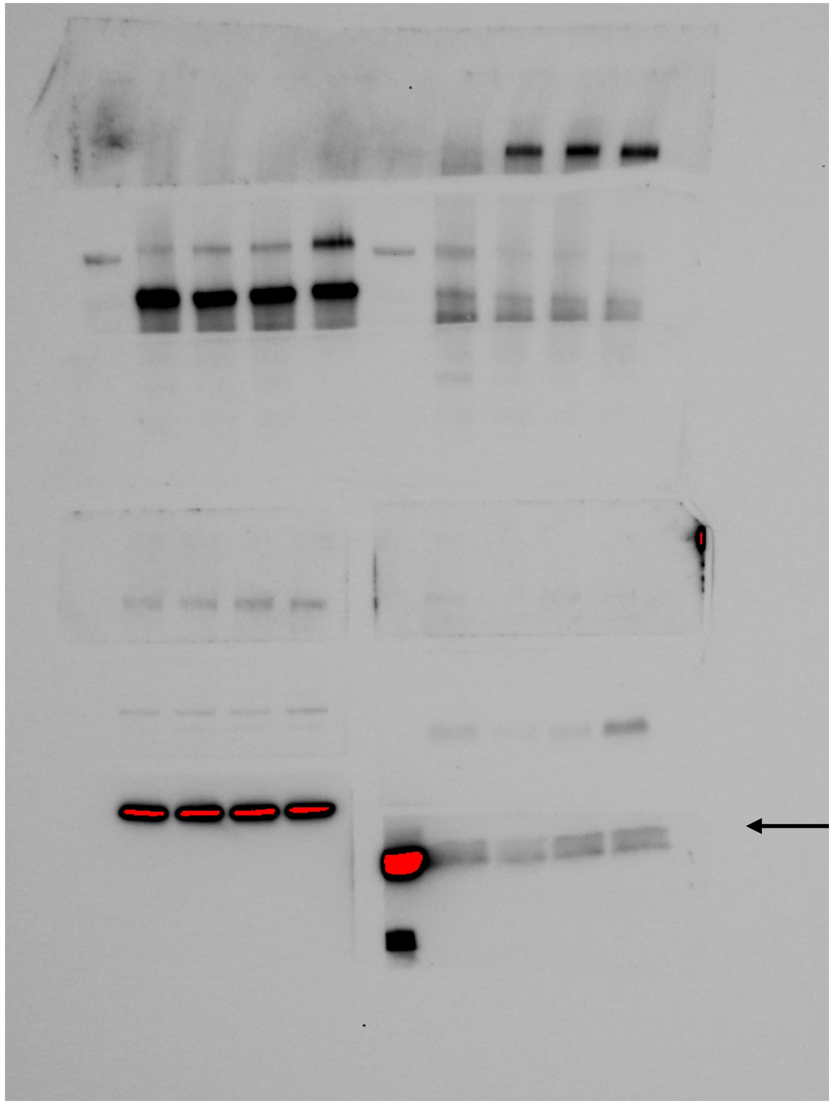

RAC-1

1 2

GAPDH

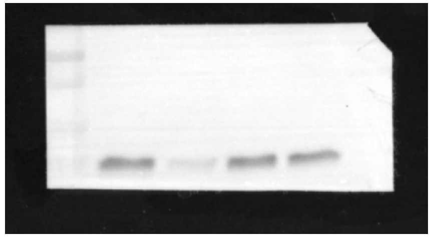

PANC-1 sgRNA system

1: PANC-1 Cas9 control

2: PANC-1 Cas9 +Dox

Supplementary Fig 4 Panel B

SMAD3

RAC-1

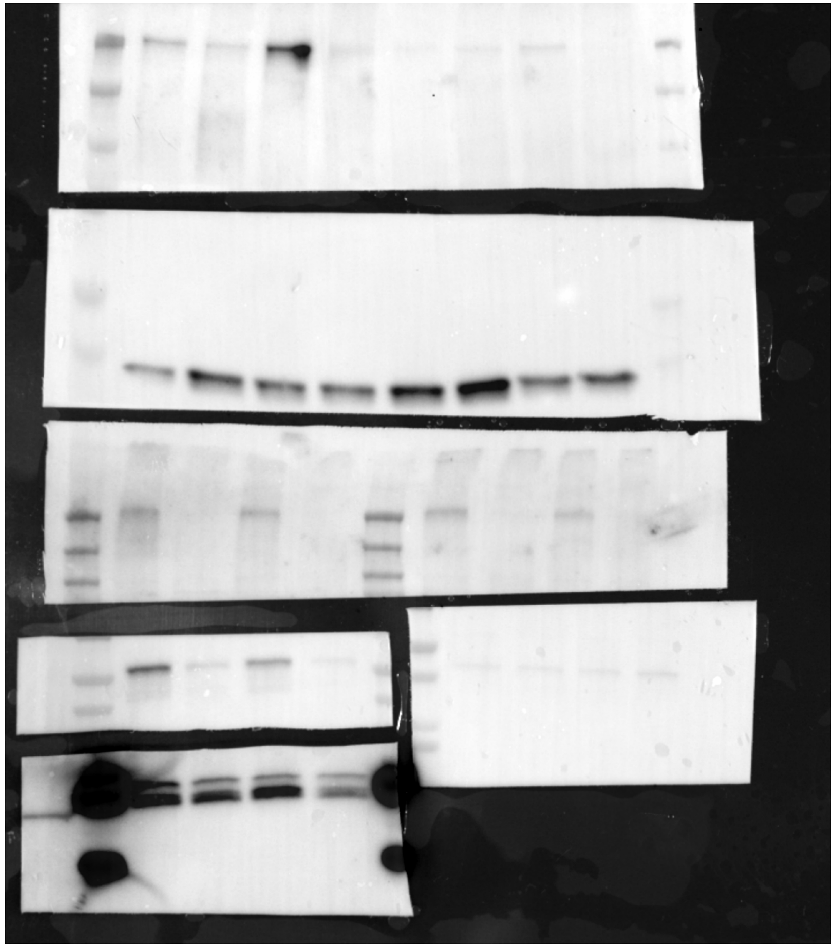

3 4

GAPDH

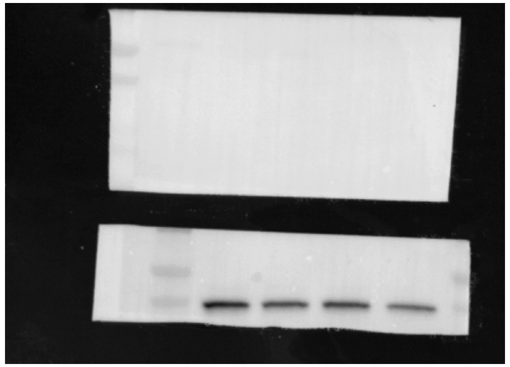

3 4

3: PANC-1 cont sh
4:PANC-1 SMAD3sh1

Supplementary Fig 4 Panel C and E

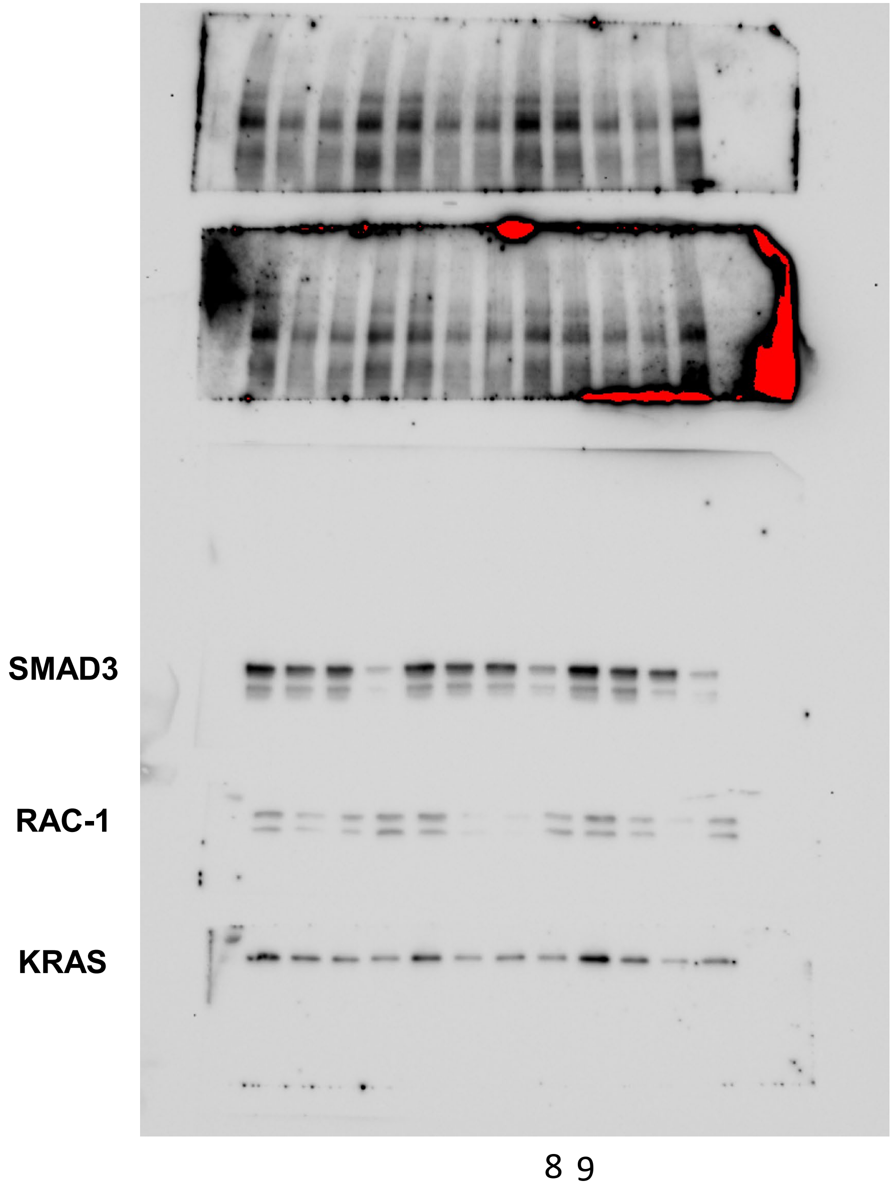

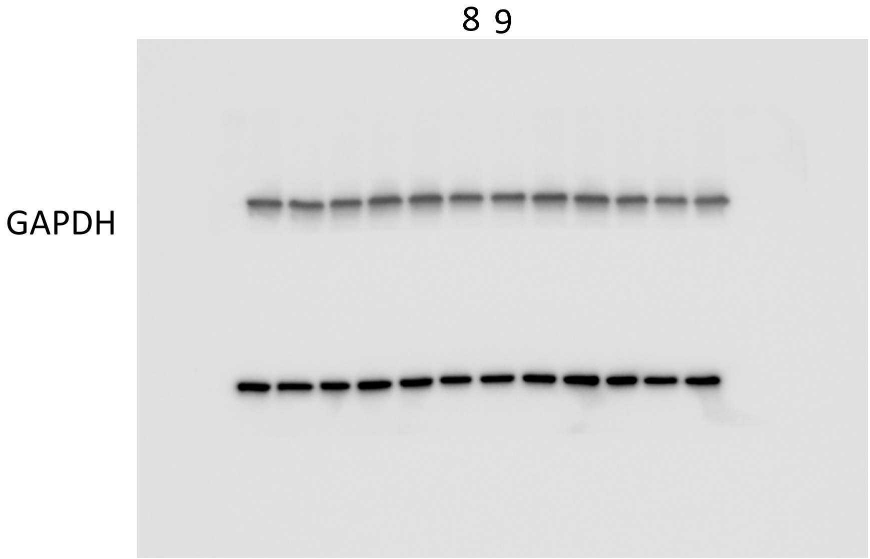

9: 4535 cont sh
8: 4535 SMAD3 sh2

Supplementary Fig 4 Panel D

SMAD3

KRAS

GAPDH

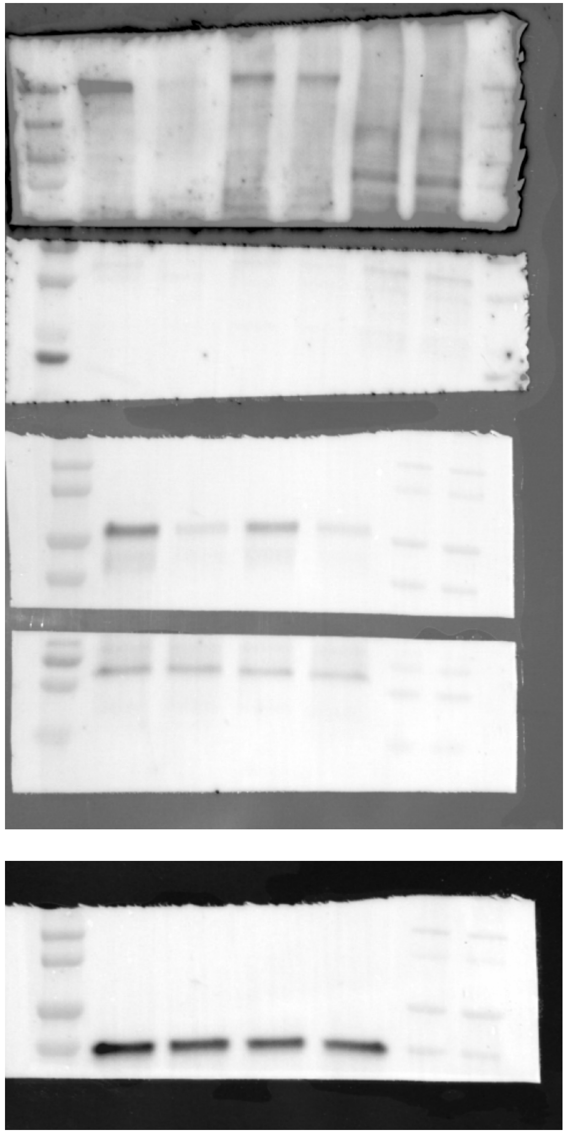

3 4

3: PANC-1 cont sh

4:PANC-1 SMAD3 sh1

1 2

SMAD3

GAPDH

KRAS

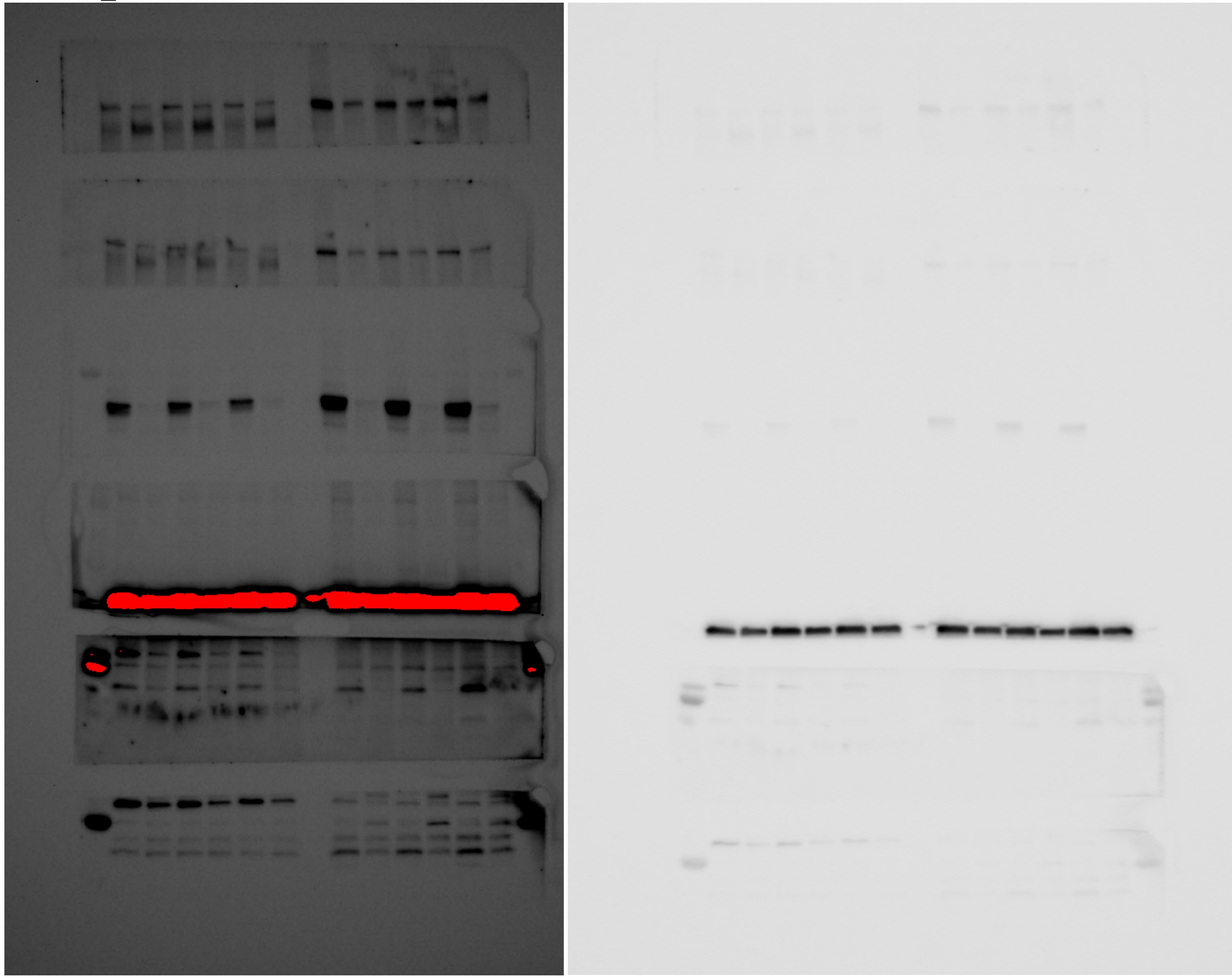

1: PANC-1 cont sh

2:PANC-1 SMAD3 sh2

Supplementary Fig 4 Panel C and E

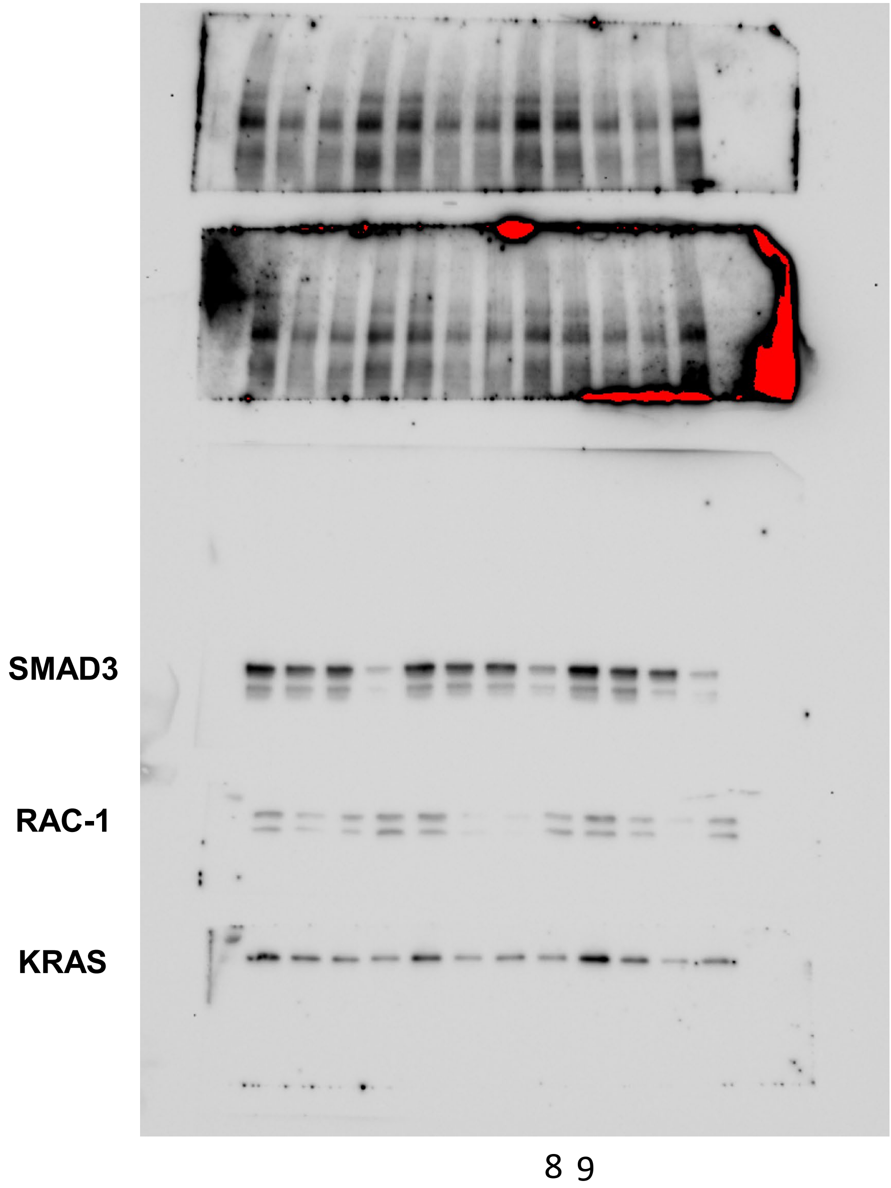

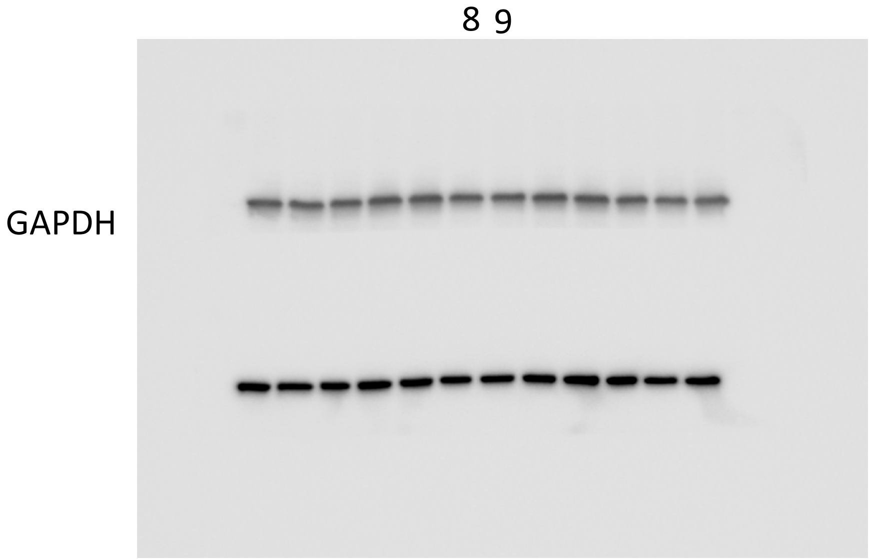

9: 4535 cont sh
8: 4535 SMAD3 sh2

Supplementary Fig 5 Panel B

7 8

PLEXIND1

E-cad

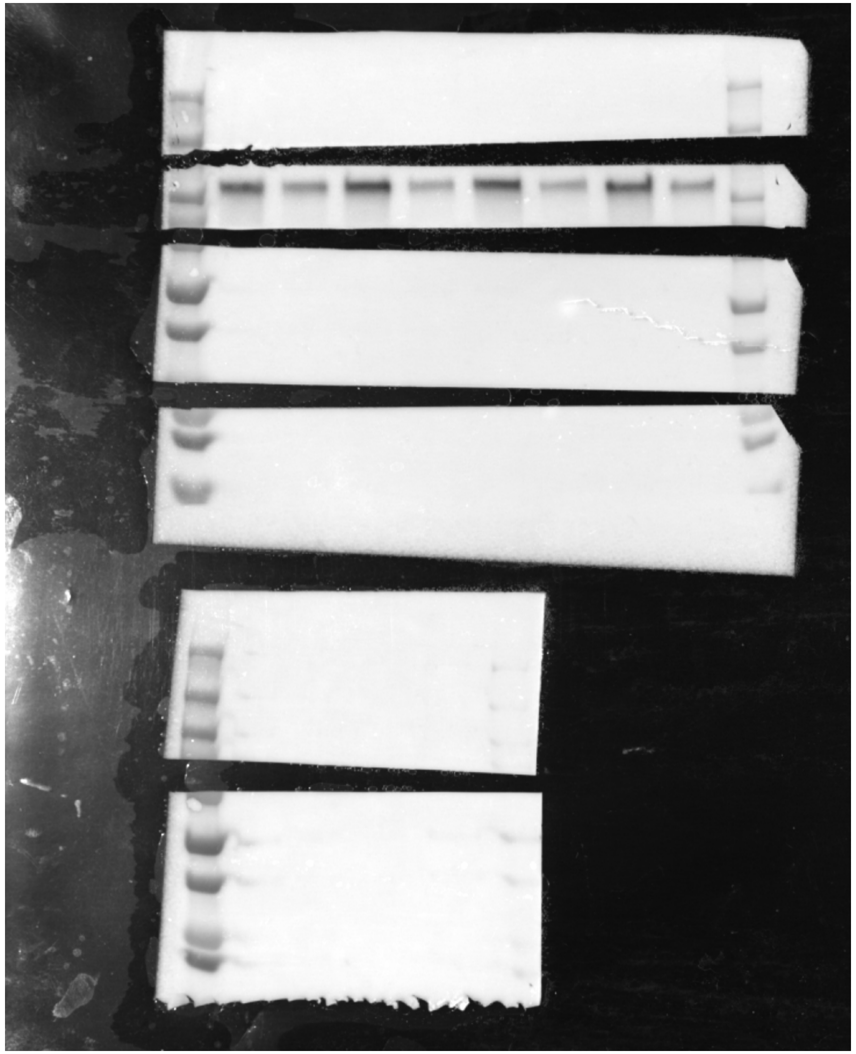

GAPDH

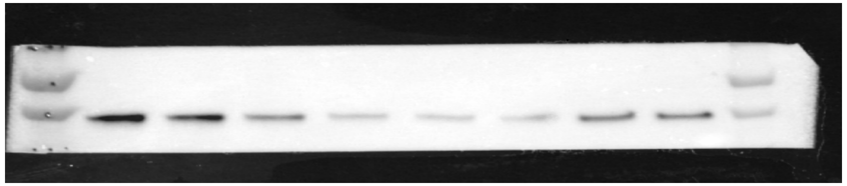

7 8

PLEXIND1

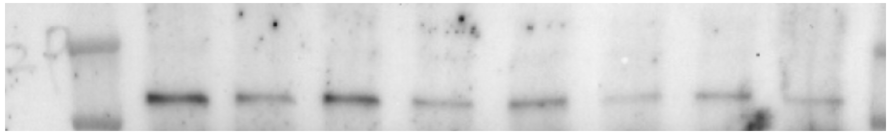

GAPDH

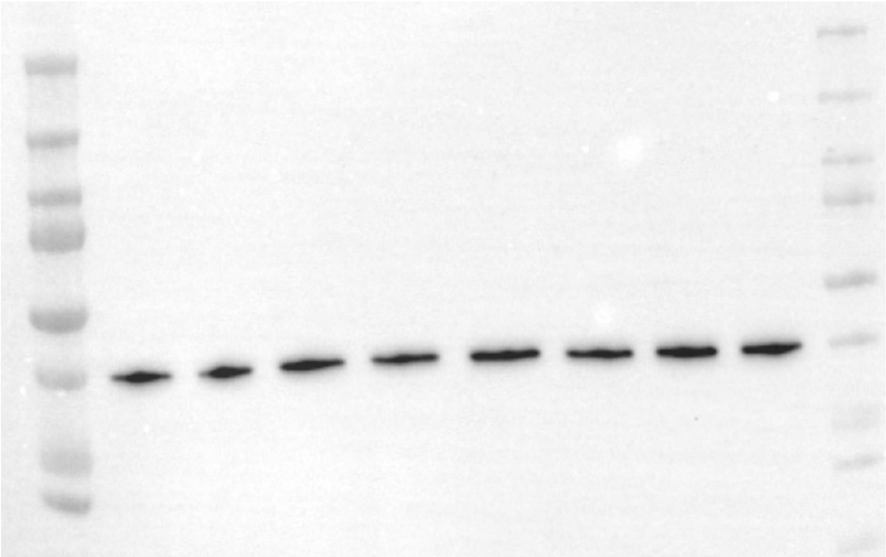

7: BxPC-3 cont sh

8:BxPC-3 PLEXIND1sh1

Supplementary Fig 5 Panel D

1 2

PLEXIND1

pSMAD3

GAPDH

SMAD3

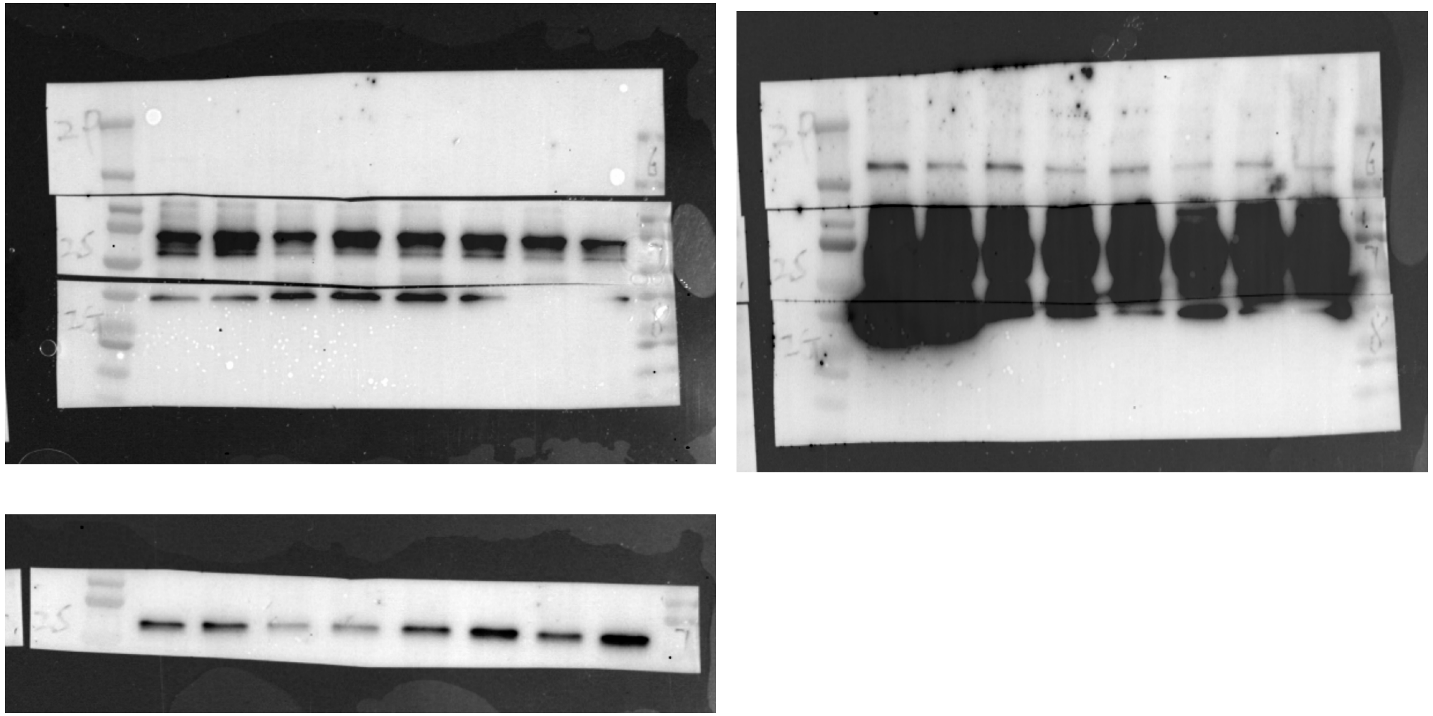

1: BxPC-3 cont sh
2:BxPC-3 PLEXIND1sh1

Supplementary Fig 5 Panel E

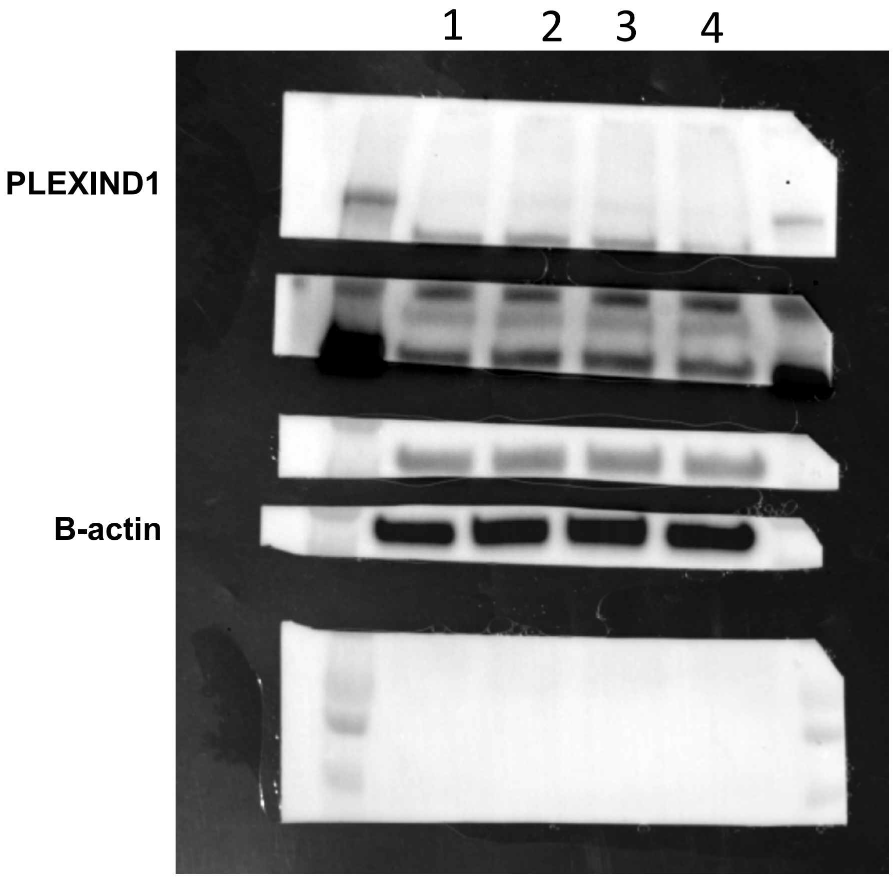

BxPC-3 Samples
1,2 : control
3- 10 mins TGFB
4-30mins TGFB

Supplementary Fig 5 Panel F

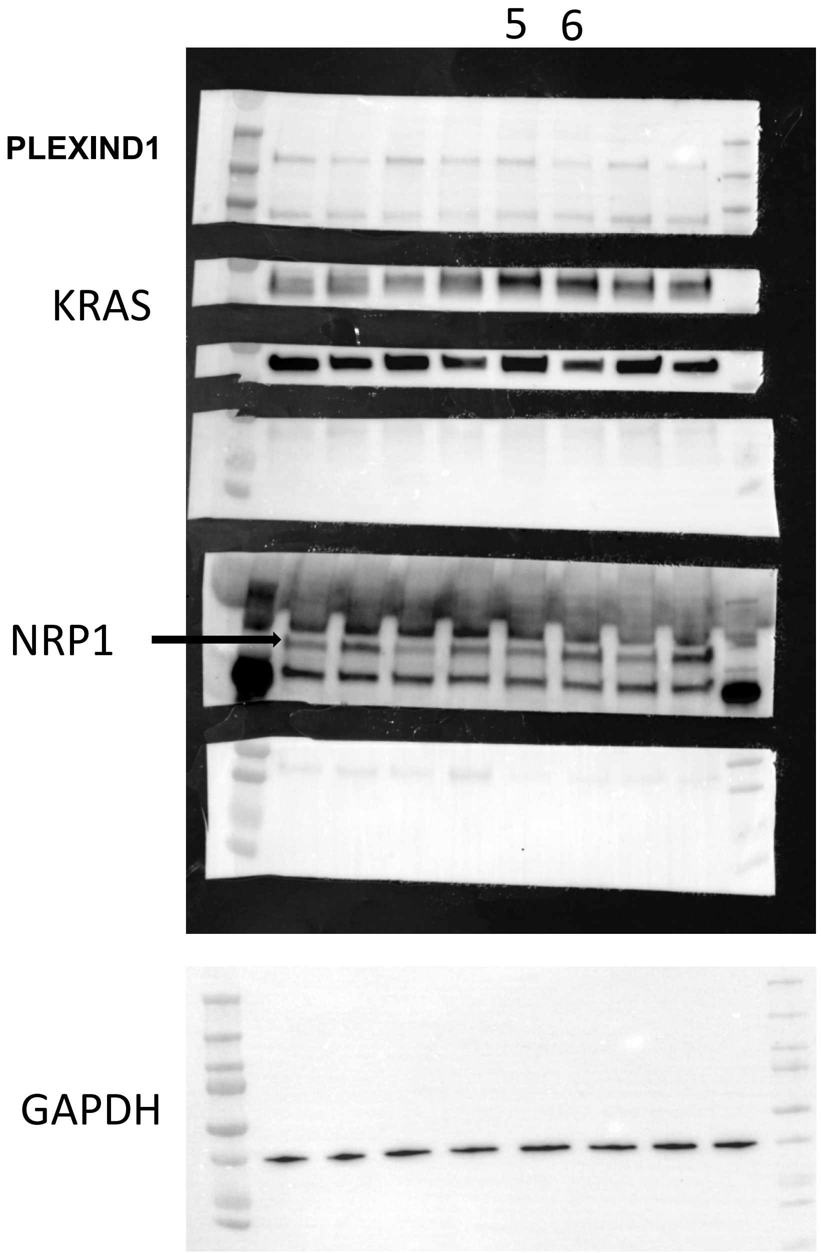

5: BxPC-3 cont sh
6:BxPC-3 PLEXIND1sh1

Supplementary Fig 5 Panel F

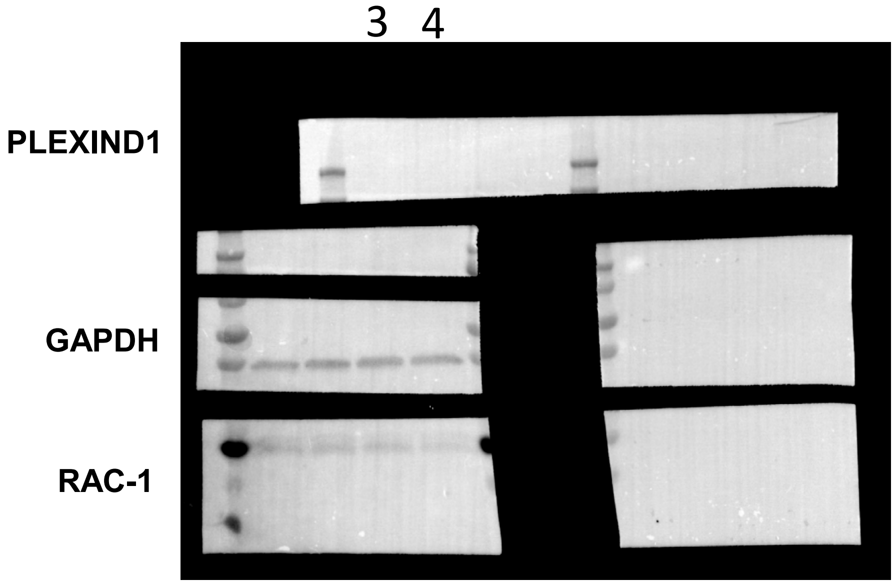

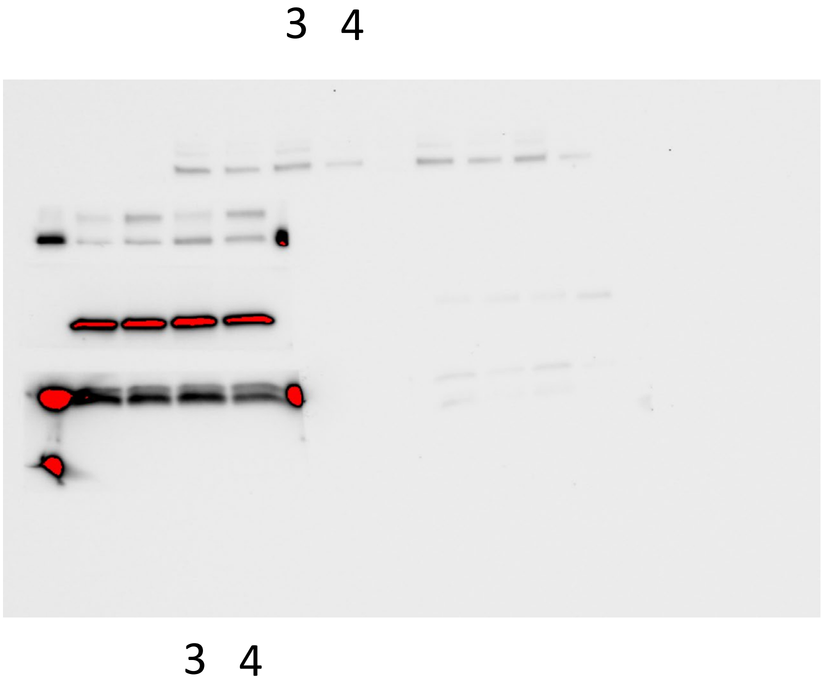

3: BxPC-3 cont sh
4:BxPC-3 PLEXIND1sh1

Supplementary Fig 5 Panel G

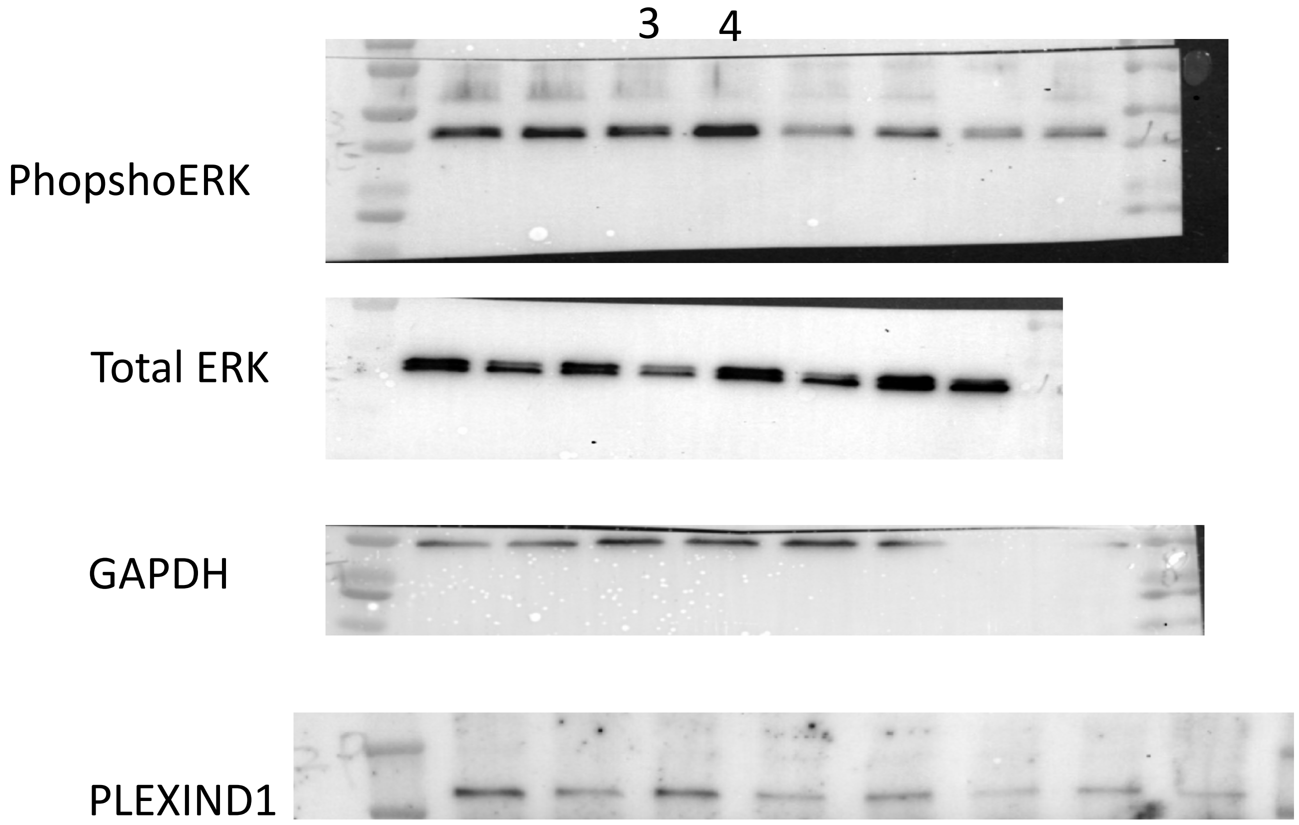

3: BxPC-3 cont sh
4:BxPC-3 PLEXIND1sh1
