# Supplementary material for: Role of PLEXIND1/TGFβ Signaling Axis in Pancreatic Ductal Adenocarcinoma Progression Correlates with the Mutational Status of KRAS
Source: Cancers (Basel). 2021 Aug 11;13(16):4048. doi: 10.3390/cancers13164048 (PMC8393884; doi:10.3390/cancers13164048)
Supplement: Supplementary file 1 [file cancers-13-04048-s001.zip › cancers-1302602 supplementary.pdf]

cancers

MDPI

Supplementary Materials: Role of PLEXIND1/TGFβ Signaling Axis in Pancreatic Ductal Adenocarcinoma Progression Correlates with the Mutational Status of *KRAS*

Sneha Vivekanandhan, Vijay S. Madamsetty, Ramcharan Singh Angom, Shamit Kumar Dutta, Enfeng Wang, Thomas Caulfield, Alexandre A. Pletnev, Rosanna Upstill-Goddard, Yan W. Asmann, David Chang, Mark R Spaller and Debabrata Mukhopadhyay

Materials and Methods:

Whole Cell Extract Preparation

Cell lines were washed three times with ice-cold PBS (pH 7.4, Gibco, Grand Island, USA) to which were added ice-cold NP-40 lysis buffer (50 mM Tris-HCl, 150 mM NaCl, 1% NP-40, and 5 mM EDTA, pH 7.4±0.2) containing 1% proteinase inhibitor cocktail (Sigma-Aldrich, St. Louis, Missouri) and 1% Halt phosphatase inhibitor cocktail (Pierce, USA) to lyse the cells. Cells were incubated on ice for thirty minutes and then centrifuged for 15 minutes at 12,000 r.p.m at 4 °C. Supernatant was collected, and protein concentration determined with the bicinchoninic acid assay (Pierce BCA Protein Assay Kit, MA, USA).

Design and synthesis of peptides

The lipidated peptides were synthesized using standard Fmoc-based solid-phase: peptide synthesis (SPPS) methods, as exemplified by the following for *N*-myristoyl-YECYSEA. Pre-loaded Fmoc-Ala-Wang resin was swollen in DMF for 30 min, and then drained. This was followed by initial Fmoc deprotection (piperidine/DMF, shaken for 1 min; drained; washed with DMF; repeated once). After DMF wash (shaken for 15 sec; drained; repeated twice), sequential coupling of the remaining residues began. This involved adding the appropriate Fmoc amino acid (5-fold excess) pre-combined with HCTU (5-fold excess) in DMF to the resin. After mixing for 20 sec, DIEA (10-fold excess) was added and allowed to react for 3 min. After DMF wash (shaken for 15 sec; drained, repeated twice) the previous piperidine deprotection conditions were used. These steps were repeated for each added amino acid. After final Fmoc deprotection, the resin was sequentially washed with DMF and DCM (twice each). Myristoylation was performed using 10-fold excess each of myristic anhydride and DIEA in DCM at r.t. overnight, followed by extensive washing with DCM. Peptide removal from the resin with global deprotection was accomplished with addition of resin cleavage solution (5x resin volume, TFA and scavengers mixture (triisopropylsilane)/thioanisole/anisole, volume ratio: [92:4:2:2]) for 1 h at r.t. The volatile components were removed under reduced pressure, the residue mixed with water and lyophilized to afford crude solid peptide. Similar procedure followed for synthesis of 8 mer of PLEXIND1 binding peptide (referred as *N*-myristoyl- YECYSEA peptide); and the scrambled peptides referred as *N*-myristoyl-AEYCESY.

Tumor growth studies

Orthotopic models were used. Mice were anesthetized, and the cells (resuspended in 100 μL of PBS) were injected into the pancreas of each mouse. For BxPC-3 cells, 1×10^6 cells treated with either control shRNA or PLEXIND1 shRNA were injected; 1×10^6 PANC-1 cells treated with either control or SMAD3 shRNA; 6×10^5 PANC-1 cells treated with either control or PLEXIND1 shRNA and 6×10^5 PANC-1 cells treated with sgRNA against PLEXIND1 were injected, respectively. The treatment group of mice injected with PANC-1 cells with sgRNA against PLEXIND1 were given doxycycline (0.5 mg/mL) throughout the

study. Upon completion of the study, the mice were sacrificed and their tumors harvested for histological analysis.

Peptide data studies

5×10^6 PANC-1 cells suspended in 100 μL PBS were injected subcutaneously into the right flanks of female SCID mice (5 mice in each group). After 30 days, the mice were randomized and either vehicle or AP1134 (dissolved in PBS containing 75% DMSO) were injected intratumorally every day from Monday to Friday for three weeks (500 μg/mouse/day). After three weeks of treatment, mice were sacrificed, and tumor growth was analyzed.

Immunohistochemical Staining

After the tumors were harvested, they were fixed in 10% neutral buffered formalin at room temperature for a day; kept overnight in 70% ethanol, then embedded in paraffin and sectioned. The sections were deparaffinized the immunohistochemical analysis performed in accordance with the manufacturers' instructions (DAB 150, Burlington MA, Millipore). Stable diaminobenzidine was used as a chromogen substrate and the sections were counterstained with a hematoxylin solution. Images were acquired using Aperio.

Structural Modeling

The sequences of human PLEXIND1 (known as Plexin-D1), TGFBRII (known as TGFβ receptor type-2), TGFB1 and TGFB2 (known as TGFβ), and NRP1 genes were modeled. The structural homologs for Monte Carlo were built and the entire full-length structure was modeled, filling in any gaps or unresolved portions. Analyses focused on the protein-protein interaction containing regions. Monte Carlo dynamics searching (MC-search) was performed on every model for additional conformation sampling using published methods [1,2,3]. The primary purpose of MC in this scenario was to examine any conformational variability that may occur with different orientations in the region near the protein-protein interfaces. The MDS biasing technique algorithm, Maxwell's demon MD, was employed to search potential flexible zones within these sites that could provide for favorable peptide interactions [4].

Protein-Protein Docking

In brief, the proteins were docked using the PIPER program within the Schrödinger software suite using a virtual screening workflow (VSW) [5]. Alternative protein-protein docking methods were also employed, including in-house software methods.

Reference

1. Caulfield T.; Medina-Franco J.L. Molecular dynamics simulations of human DNA methyltransferase 3B with selective inhibitor nanaomycin. *A. J Struct Biol.* **2011**, *176*, 185–91.
2. Caulfield T.R. Inter-ring rotation of apolipoprotein A-I protein monomers for the double-belt model using biased molecular dynamics. *J Mol Graph Model.* **2011**, *29*, 1006–14.
3. Caulfield T.; Devkota B. Motion of transfer RNA from the A/T state into the A-site using docking and simulations. *Proteins*. **2012**, 80, 2489–500.
4. Caulfield T.R.; Devkota B.; Rollins G.C. Examinations of tRNA Range of Motion Using Simulations of Cryo-EM Microscopy and X-Ray Data. *J Biophys*. **2011**, *2011*: 219515.
5. Bhachoo J., Beuming T. Investigating Protein-Peptide Interactions Using the Schrodinger Computational Suite. *Methods Mol Biol.* **2017**, *1561*, 235–254.

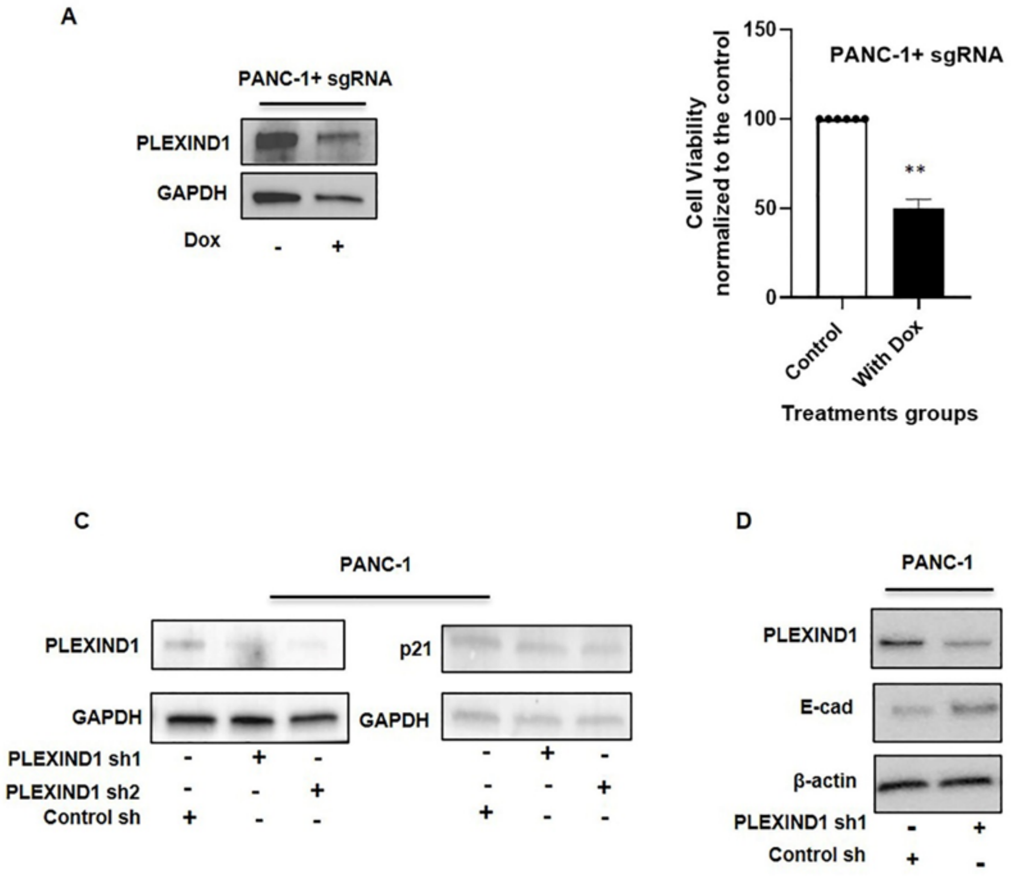

Figure S1. Functional role of PLEXIND1 expression in different PDAC cell lines. (**A**): Western blot analysis showing reduced expression PLEXIND1 in PANC-1 cells after sgRNA treatment. (**B**): Cell viability assay for PANC-1 cells with and without sgRNA mediated PLEXIND1 knockdown grown in 2D cell culture for 72 hours. Data is plotted as percentage of control cells. (**C,D**): Western blot analysis showing expression of p21 and E-cadherin in PANC-1 cells with reduced PLEXIND1 levels. Statistical significance *p <0.05 vs control ** p<. 01 vs control. Error bars indicate standard error of mean.

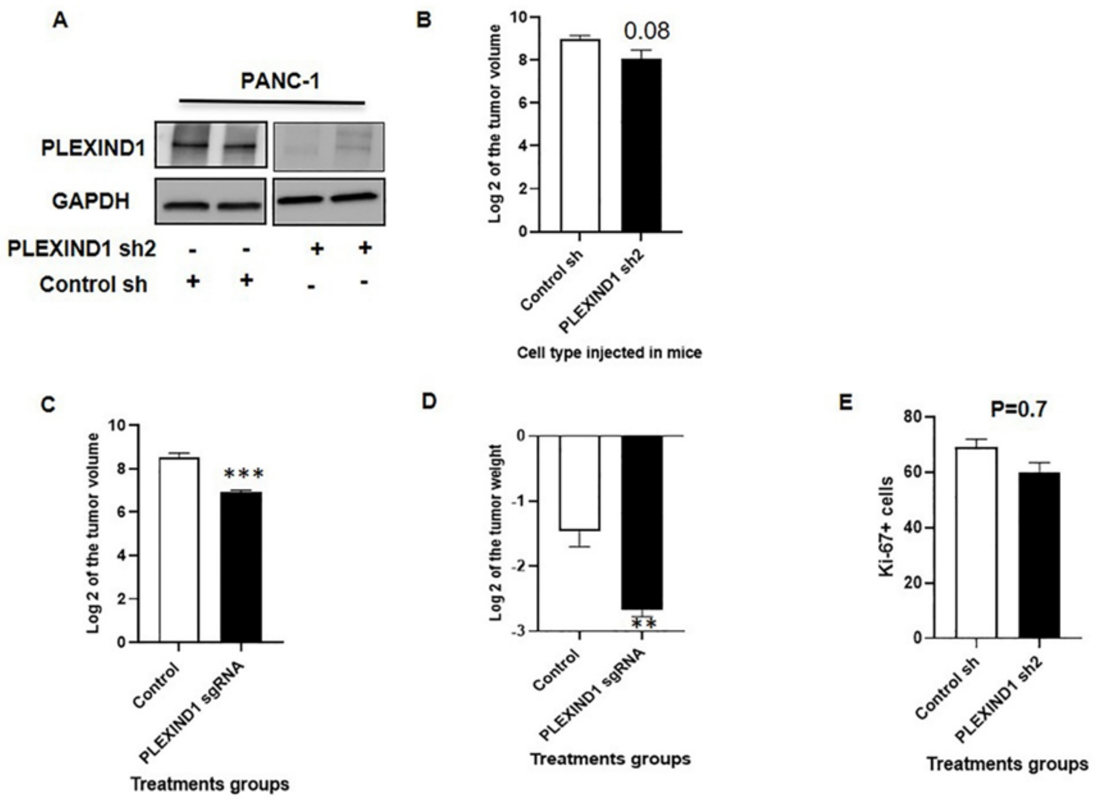

Figure S2. Role of PLEXIND1 in PDAC progression. (**A**): Western blot analysis showing reduced expression PLEXIND1 in tissues from orthotropic mice models implanted with PANC-1 cells with PLEXIND1 knockdown (**B**): Log2 values of tumor volumes from orthotropic mice models implanted with PANC-1 cells treated with control or PLEXIND1 shRNA2. (**C–D**): Log2 values of tumor volumes and weights from mice implanted with PANC-1 cells with PLEXIND1 knockdown mediated by sgRNA. E: Quantification of digital images of Ki-67 staining of tumor tissues obtained from mice implanted with PANC-1 cells treated with control and PLEXIND1 shRNA2. Statistical significance: *p<0.05 vs. control group, **p<0.01 vs. control group, ***p<0.001 vs. control group . Error bars indicate standard error of the mean.

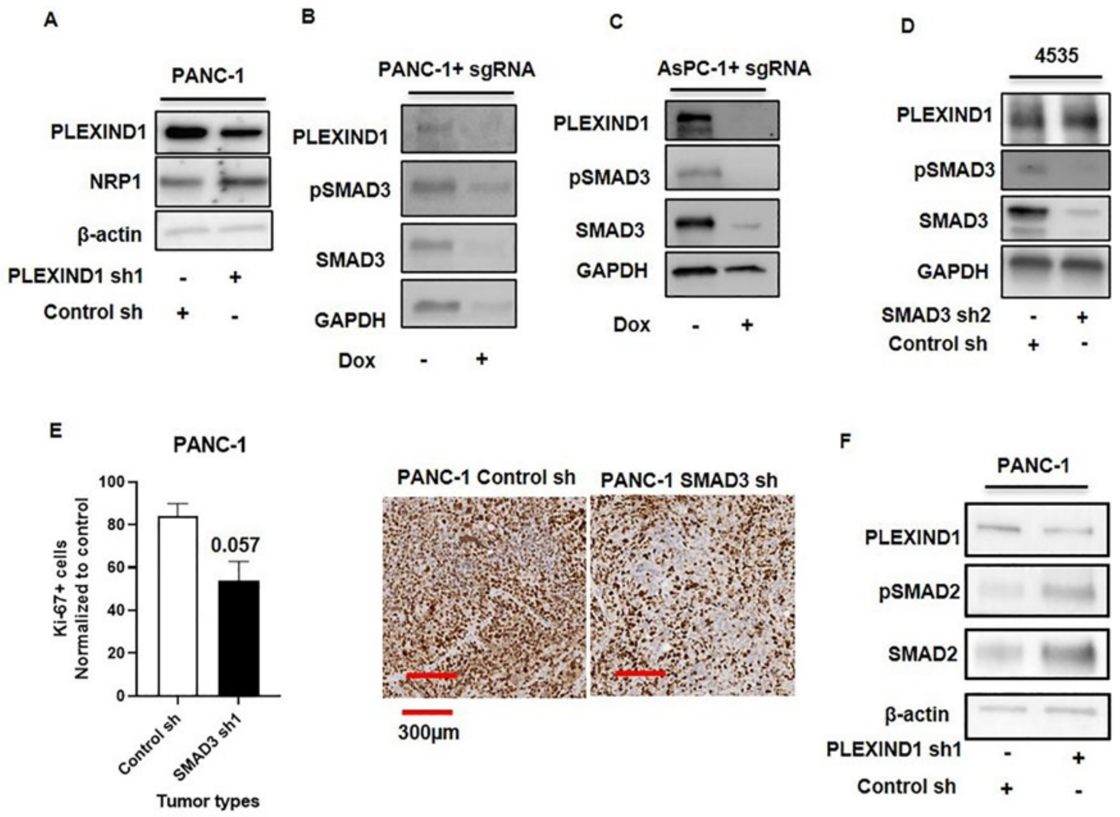

Figure S3. Reduced PLEXIND1 expression in PDAC modulates tumor growth through SMAD3 signaling. (**A**): Western blot analysis showing expression of NRP1 in PANC-1 cells with reduced PLEXIND1 levels. (**B–C**): Western blot analysis showing levels of phosphorylated and total SMAD3 in PANC-1 and AsPC-1 cells with PLEXIND1 knockdown mediated by sgRNA. (**D**): Western blot analysis showing levels of PLEXIND1, phosphorylated and total SMAD3 in 4535 cells after SMAD3 knockdown. (**E**): Representative images and quantification of digital images for Ki-67 staining at 20X magnification. (**F**): Western blot analysis showing levels of phosphorylated and total SMAD2 in PANC-1 cells with decreased PLEXIND1 levels. Statistical significance: *p<0.05 vs. control group, **p<0.01 vs. control group. Error bars indicate standard error of the mean.

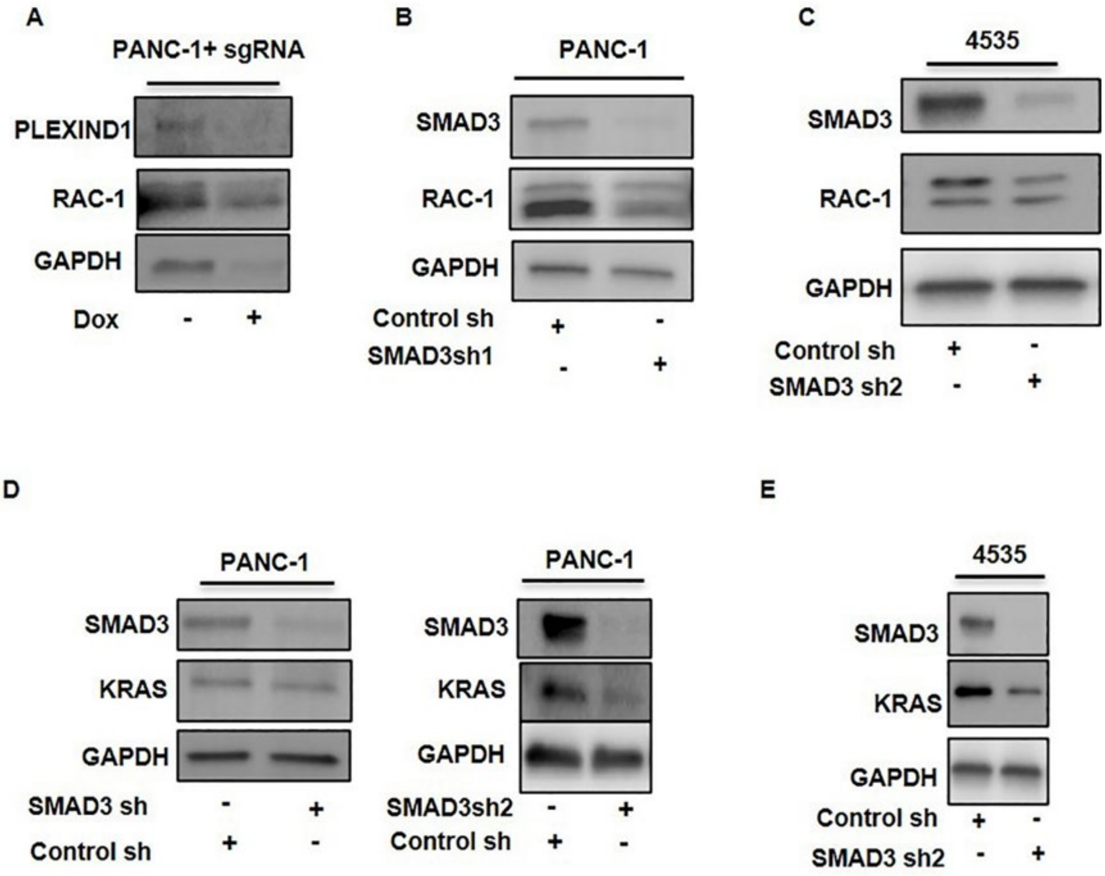

Figure S4. Decreased PLEXIND1 expression reduces RAC-1 expression in PDAC cell lines. (**A**):Western blot analysis showing protein expression of RAC-1 in PANC-1 cells with PLEXIND1 knockdown mediated by sgRNA. (**B–C**): Western blot analysis showing expression of RAC-1 in PANC-1 and 4535 cells with SMAD3 knockdown. (**D–E**): KRAS protein expression in PANC-1 and 4535 cells with reduced SMAD3 levels.

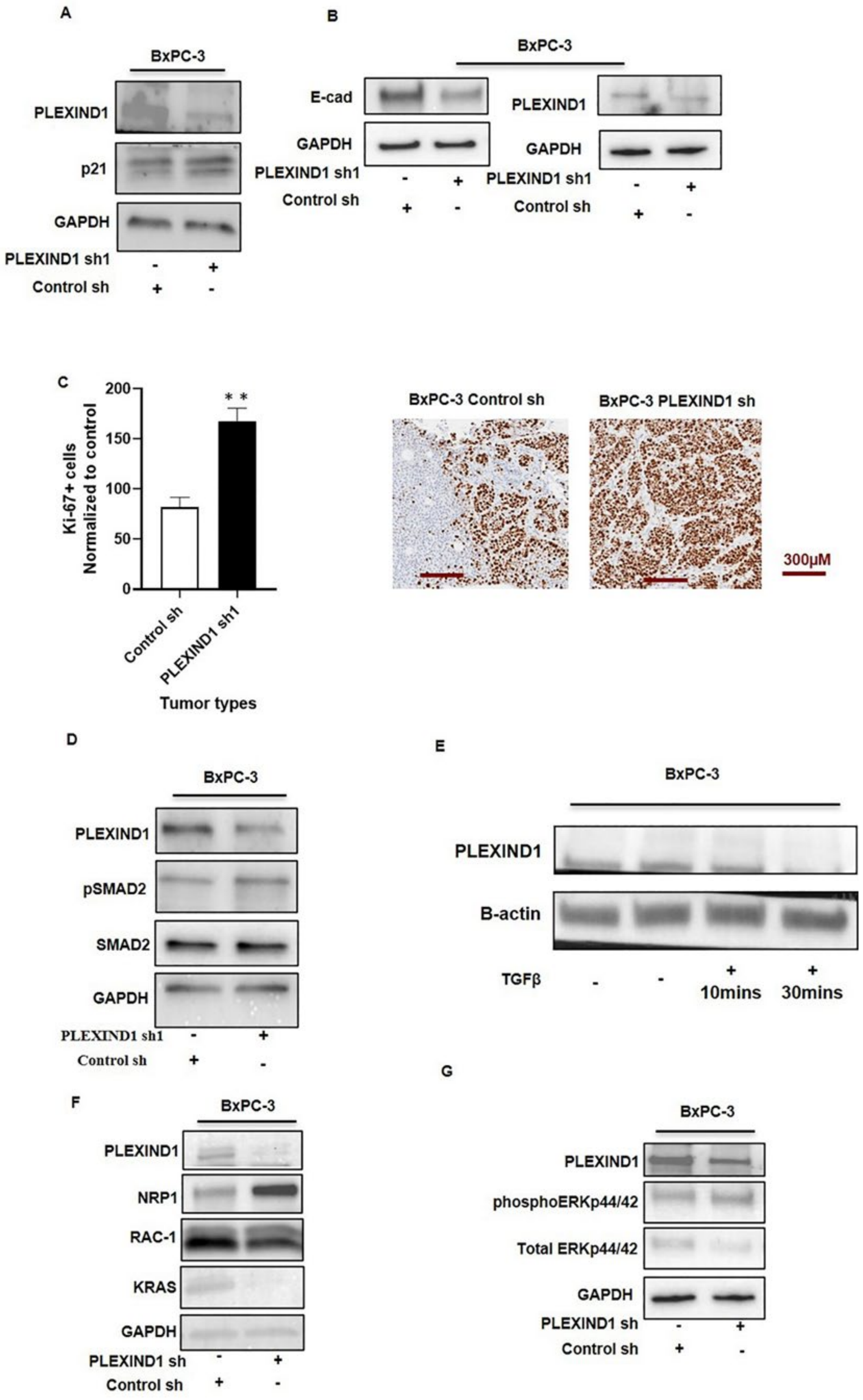

Figure S5. PLEXIND1 acts as a Tumor Suppressor in KRASwt PDAC cell line. (**A–B**): Western blot analysis showing expression of p21 and E-cad in BxPC-3 cells with reduced PLEXIND1 levels. (**C**): Representative images and quantification of digital images for Ki-67 staining at 20X magnification. (**D**): Western blot analysis showing levels of phosphorylated and

total SMAD2 in BxPC-3 cells with decreased PLEXIND1 levels. E: Western blot assay showing changes in PLEXIND1 expression in BxPC-3 cells post TGFβ induction. (**F–G**): Protein expressions of NRP1, RAC-1, KRAS, phosphorylated, and total ERK p44/42. Error bars indicate standard error of the mean.

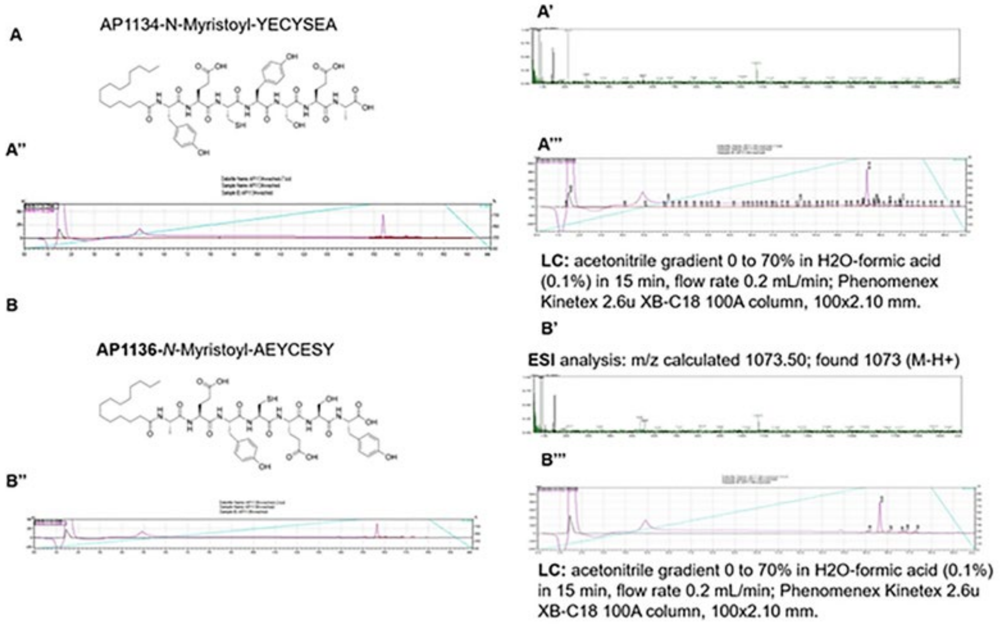

Figure S6. Therapeutic potential of peptide-mediated targeting of PLEXIND1. A-A′′′; B-B′′′: Structural representation of PLEXIND1-based peptides.

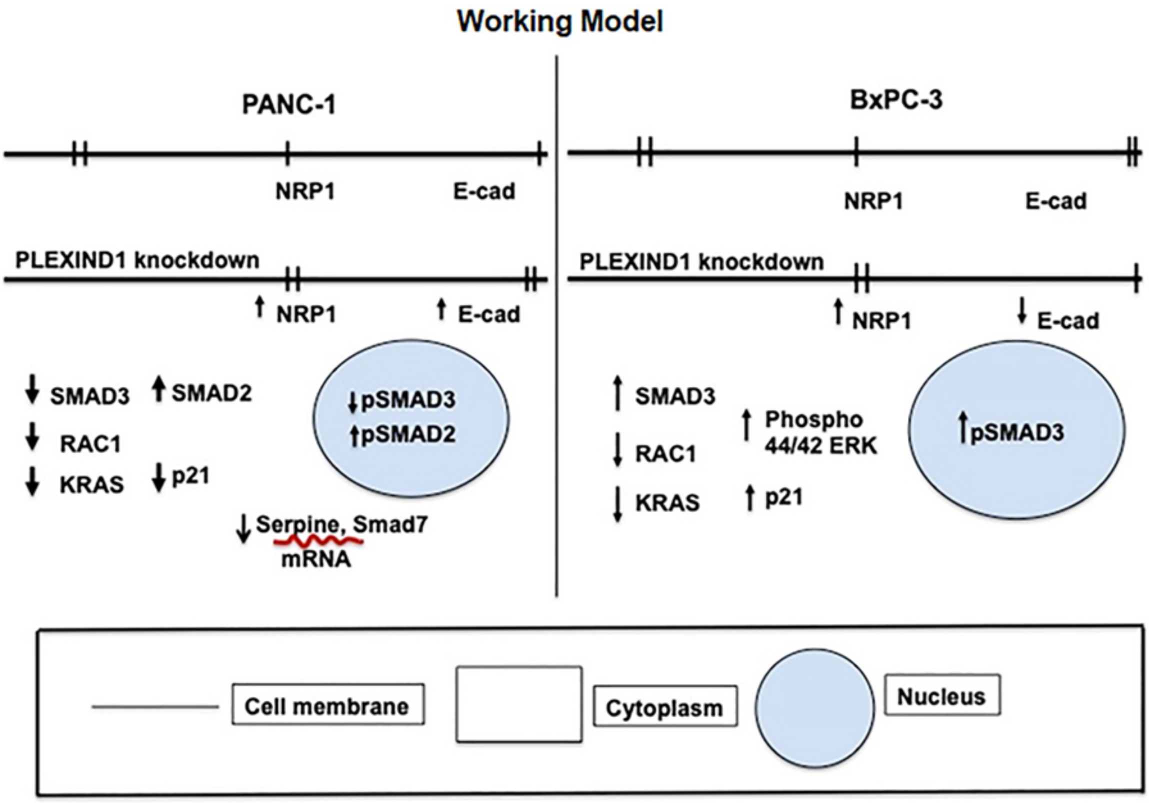

Figure S7. Working model. Our working model on how PLEXIND1 mediates TGFβ signaling in PANC-1 (KRASmt) and BxPC-3 (KRASwt) cells.
